# Contributions of common genetic variants to risk of schizophrenia among individuals of African and Latino ancestry

**DOI:** 10.1038/s41380-019-0517-y

**Published:** 2019-10-07

**Authors:** Tim B. Bigdeli, Giulio Genovese, Penelope Georgakopoulos, Jacquelyn L. Meyers, Roseann E. Peterson, Conrad O. Iyegbe, Helena Medeiros, Jorge Valderrama, Eric D. Achtyes, Roman Kotov, Eli A. Stahl, Colony Abbott, Maria Helena Azevedo, Richard A. Belliveau, Elizabeth Bevilacqua, Evelyn J. Bromet, William Byerley, Celia Barreto Carvalho, Sinéad B. Chapman, Lynn E. DeLisi, Ashley L. Dumont, Colm O’Dushlaine, Oleg V. Evgrafov, Laura J. Fochtmann, Diane Gage, James L. Kennedy, Becky Kinkead, Antonio Macedo, Jennifer L. Moran, Christopher P. Morley, Mantosh J. Dewan, James Nemesh, Diana O. Perkins, Shaun M. Purcell, Jeffrey J. Rakofsky, Edward M. Scolnick, Brooke M. Sklar, Pamela Sklar, Jordan W. Smoller, Patrick F. Sullivan, Fabio Macciardi, Stephen R. Marder, Ruben C. Gur, Raquel E. Gur, David L. Braff, Consortium on the Genetics of Schizophrenia (COGS) Investigators, Monica E. Calkins, Robert R. Freedman, Michael F. Green, Tiffany A. Greenwood, Laura C. Lazzeroni, Gregory A. Light, Keith H. Nuechterlein, Allen D. Radant, Larry J. Seidman, Larry J. Siever, Jeremy M. Silverman, William S. Stone, Catherine A. Sugar, Neal R. Swerdlow, Debby W. Tsuang, Ming T. Tsuang, Bruce I. Turetsky, Humberto Nicolini, Michael A. Escamilla, Marquis P. Vawter, Janet L. Sobell, Dolores Malaspina, Douglas S. Lehrer, Peter F. Buckley, Mark H. Rapaport, James A. Knowles, Genomic Psychiatry Cohort (GPC) Consortium, Ayman H. Fanous, Michele T. Pato, Steven A. McCarroll, Carlos N. Pato

**Affiliations:** 1 Department of Psychiatry and Behavioral Sciences, SUNY Downstate Medical Center, Brooklyn, NY USA; 2 Institute for Genomic Health, SUNY Downstate Medical Center, Brooklyn, NY USA; 3 Department of Psychiatry, Veterans Affairs New York Harbor Healthcare System, Brooklyn, NY USA; 4 Stanley Center for Psychiatric Research, Broad Institute of MIT and Harvard, Cambridge, MA USA; 5 Department of Genetics, Harvard Medical School, Boston, MA USA; 6 Department of Psychiatry, Virginia Commonwealth University, Richmond, VA USA; 7 Department of Psychosis Studies, King’s College London, London, UK; 8 Cherry Health and Michigan State University College of Human Medicine, Grand Rapids, MI USA; 9 Department of Psychiatry, Stony Brook University, Stony Brook, NY USA; 10 Department of Psychiatry, Icahn School of Medicine at Mount Sinai, Mount Sinai, NY USA; 11 Department of Genetics & Genomics, Icahn School of Medicine at Mount Sinai, Mount Sinai, NY USA; 12 Department of Psychiatry & Behavioral Sciences, University of Southern California, Los Angeles, CA USA; 13 Institute of Medical Psychology, Faculty of Medicine, University of Coimbra, Coimbra, PT Portugal; 14 Beacon Health Options, Boston, MA USA; 15 Department of Psychiatry, University of California, San Francisco, CA USA; 16 Faculty of Social and Human Sciences, University of Azores, Ponta Delgada, Portugal; 17 VA Boston Healthcare System, Brockton, MA USA; 18 Department of Psychiatry, Harvard Medical School, Boston, MA USA; 19 Department of Cell Biology, SUNY Downstate Medical Center, Brooklyn, NY USA; 20 Neurogenetics Laboratory, Campbell Family Mental Health Research Institute, Centre for Addiction and Mental Health; Department of Psychiatry, University of Toronto, Toronto, ON Canada; 21 Department of Psychiatry and Behavioral Sciences, Emory University, Atlanta, GA USA; 22 Department of Public Health and Preventive Medicine, State University of New York, Upstate Medical University, Syracuse, NY USA; 23 Department of Family Medicine, State University of New York, Upstate Medical University, Syracuse, NY USA; 24 Department of Psychiatry and Behavioral Sciences, State University of New York, Upstate Medical University, Syracuse, NY USA; 25 Department of Psychiatry, University of North Carolina, Chapel Hill, NC USA; 26 Department of Psychiatry, Brigham and Women’s Hospital, Boston, MA USA; 27 Department of Psychiatry, Massachusetts General Hospital, Boston, MA USA; 28 Department of Epidemiology, Harvard T.H. Chan School of Public Health, Boston, MA USA; 29 Medical Epidemiology and Biostatistics, Karolinska Institutet, Solna, SE Sweden; 30 Department of Psychiatry and Human Behavior, University of California, Irvine, CA USA; 31 Department of Psychiatry and Biobehavioral Sciences, Geffen School of Medicine, University of California Los Angeles, Los Angeles, CA USA; 32 Semel Institute for Neuroscience and Human Behavior, Geffen School of Medicine, University of California Los Angeles, Los Angeles, CA USA; 33 Department of Psychiatry, University of Pennsylvania Perelman School of Medicine and Children’s Hospital of Philadelphia, Philadelphia, PA USA; 34 Child & Adolescent Psychiatry, University of Pennsylvania Perelman School of Medicine and Children’s Hospital of Philadelphia, Philadelphia, PA USA; 35 Lifespan Brain Institute, University of Pennsylvania Perelman School of Medicine and Children’s Hospital of Philadelphia, Philadelphia, PA USA; 36 Department of Psychiatry, University of California, La Jolla, San Diego, CA USA; 37 VISN-22 Mental Illness, Research, Education and Clinical Center (MIRECC), VA San Diego Healthcare System, San Diego, CA USA; 38 Carracci Medical Group, Mexico City, MX Mexico; 39 Department of Psychiatry, Texas Tech University Health Sciences Center, El Paso, TX USA; 40 Department of Psychiatry, Wright State University, Dayton, OH USA; 41 School of Medicine, Virginia Commonwealth University, Richmond, VA USA; 42 Department of Psychiatry, University of Colorado Denver, Aurora, CO USA; 43 VA Greater Los Angeles Healthcare System, Los Angeles, CA USA; 44 Department of Psychiatry and Behavioral Sciences, Stanford University, Stanford, CA USA; 45 Department of Pediatrics, Stanford University, Stanford, CA USA; 46 Department of Psychiatry and Behavioral Sciences, University of Washington, Seattle, WA USA; 47 VA Puget Sound Health Care System, Seattle, WA USA; 48 Massachusetts Mental Health Center Public Psychiatry Division of the Beth Israel Deaconess Medical Center, Boston, MA USA; 49 James J. Peters Veterans Affairs Medical Center, Bronx, NY USA; 50 Department of Biostatistics, University of California Los Angeles School of Public Health, Los Angeles, CA USA; 51 Institute for Genomic Medicine, University of California San Diego, La Jolla, CA USA; 52 Harvard Institute of Psychiatric Epidemiology and Genetics, Boston, MA USA

**Keywords:** Genetics, Schizophrenia

## Abstract

Schizophrenia is a common, chronic and debilitating neuropsychiatric syndrome affecting tens of millions of individuals worldwide. While rare genetic variants play a role in the etiology of schizophrenia, most of the currently explained liability is within common variation, suggesting that variation predating the human diaspora out of Africa harbors a large fraction of the common variant attributable heritability. However, common variant association studies in schizophrenia have concentrated mainly on cohorts of European descent. We describe genome-wide association studies of 6152 cases and 3918 controls of admixed African ancestry, and of 1234 cases and 3090 controls of Latino ancestry, representing the largest such study in these populations to date. Combining results from the samples with African ancestry with summary statistics from the Psychiatric Genomics Consortium (PGC) study of schizophrenia yielded seven newly genome-wide significant loci, and we identified an additional eight loci by incorporating the results from samples with Latino ancestry. Leveraging population differences in patterns of linkage disequilibrium, we achieve improved fine-mapping resolution at 22 previously reported and 4 newly significant loci. Polygenic risk score profiling revealed improved prediction based on trans-ancestry meta-analysis results for admixed African (Nagelkerke’s *R*^2^ = 0.032; liability *R*^2^ = 0.017; *P* < 10^−52^), Latino (Nagelkerke’s *R*^2^ = 0.089; liability *R*^2^ = 0.021; *P* < 10^−58^), and European individuals (Nagelkerke’s *R*^2^ = 0.089; liability *R*^2^ = 0.037; *P* < 10^−113^), further highlighting the advantages of incorporating data from diverse human populations.

## Introduction

Schizophrenia is a common (~0.6–1%), chronic and debilitating neuropsychiatric syndrome for which most of the variability in liability is attributable to genetic factors (~80%) [1]. While rare genetic variants play a role in the underlying liability [2–11], most of the currently explained liability is harbored in common variation [7, 12–14]. Genome-wide common variants, routinely assayed by commercially available genotyping arrays, can explain up to 20% of the variability in liability to schizophrenia, but its multifactorial architecture is highly complex, and the strongest associations from large GWAS of schizophrenia account collectively for only 6.2% of the explainable heritability in individuals of European descent [15].

The past decade has seen the successes of psychiatric GWAS abound, including the first definitive demonstration of polygenic influences on schizophrenia risk and its shared basis with bipolar disorder [14], and ever-increasing numbers of robustly associated, replicated SNP associations, culminating in the identification of 108 physically distinct risk loci for schizophrenia [12], a number which has since grown to 145 [16]. This progress can be credited to collaborative enterprise on an unprecedented scale, as exemplified by the Psychiatric Genomics Consortium (PGC), and a philosophy of data sharing that has enabled widespread meta-analysis and replication [17].

The largest genome-wide association studies (GWAS) have disproportionately focused on cohorts of European descent [12, 14, 18, 19]. This European bias is not unique to psychiatric genetics research, but systemic within the GWAS literature. Although the proportion of non-Europeans has since increased to ~20%, this is primarily due to greater representation of Asian populations [20, 21]. Importantly, empirical evidence indicates that at least some of this common variant attributable risk is shared between populations of European, East-Asian and African ancestry [14, 22, 23], suggesting that variation predating divergence of European and African populations harbors most of the heritability of schizophrenia.

To our knowledge, the largest schizophrenia cohort of African ancestry that has been genotyped is the Molecular Genetics of Schizophrenia (MGS-AA) study (*N* = 2259 African-American individuals) [14, 24, 25]. In the first definitive demonstration of polygenic influences on schizophrenia risk, aggregate genetic scores constructed from International Schizophrenia Consortium GWAS results were of significant but attenuated predictive value in African-ancestry individuals—individual-level scores predicted ~2–3% of the variance in schizophrenia risk in European samples, and less than half a percent in MGS-AA [14]. It is by now well understood that both specific and aggregate GWAS findings are incompletely generalizable across diverse populations [26–28], owing largely to population differences in genome-wide allele frequencies and patterns of linkage disequilibrium [29, 30].

We have undertaken the largest GWAS of admixed African individuals to date, with a combined sample size of 6152 schizophrenia and schizoaffective disorder cases and 3918 screened controls from the Genomic Psychiatry Cohort (GPC). With available sample sizes now on a par with earlier European GWAS that yielded the first replicated, genome-wide significant associations with single nucleotide polymorphisms (SNP), we consider evidence of novel genetic associations and assess trans-ancestry replication support for 128 independent associations (representing 108 physical loci) identified in the landmark study of the Psychiatric Genomics Consortium Schizophrenia Working Group (PGC-SCZ2). We consider the implications of the pronounced underrepresentation of African and Latino populations in psychiatric GWAS and highlight the potential for improved fine-mapping resolution at identified risk loci by incorporating data from diverse populations.

## Methods

### Subject ascertainment and diagnosis

The GPC is a large cosmopolitan sample of repository and newly ascertained schizophrenia and bipolar disorder cases and screened controls, with considerable representation of individuals with African, European, and Latino ancestries. In the present analysis, we considered as cases all individuals with a diagnosis of schizophrenia or schizoaffective disorder. Details of ascertainment and diagnosis are given in the Supplemental Material.

### Single nucleotide polymorphism (SNP) genotyping and imputation

Genotyping of *N* *=* 33,422 participants was performed on Illumina Infinium arrays in a total of 11 “batches” (Table 1); four of these cohorts were ascertained as being primarily of African ancestry (OmniExpress 2.5 and Multi-Ethnic Global Array); three cohorts were of broadly Latino background (OmniExpress 2.5 and Multi-Ethnic Global Array); one included participants of any background (Global Screening Array); and three consisted mainly of European participants (OmniExpress and PsychArray) selected as part of parallel research initiatives. Typed variants were aligned to the human reference genome (GRCh37). Within each genotyping batch, we excluded any variant with missingness greater than 2% or Hardy−Weinberg Equilibrium *P* value <10^−6^. Our scripts for pre-processing GWAS array data are downloadable from https://github.com/freeseek/gwaspipeline.

**Table 1:** GPC sample sizes by genotyping batch and assigned ancestry. For constituent datasets in the current analysis (Genotyping Wave/Batch), the commercial genotyping array and the numbers of individuals assigned to African, Latino, and European ancestry groups are displayed. Within each ancestry group, the reported total is based on those quantities appearing in boldface

Genotyping Wave/Batch	Illumina SNP array	Assigned ancestry					
		African (admixed)		Latino (admixed)		European	
		Cases	Controls	Cases	Controls	Cases	Controls
GPC-AA w1	Omni2.5	**1737**	**841**	5	10	28	21
GPC-AA w2	Omni2.5	**638**	**406**	0	0	10	7
GPC-Latino w1	Omni2.5	**99**	**63**	**634**	**1743**	**218**	**224**
GPC-Latino w2	Omni2.5	**55**	**29**	**115**	**741**	**118**	**145**
GPC-Global	Global Screening Array	**1233**	**1110**	**148**	**180**	**627**	**530**
COGS-AA	Multi-ethnic Global	**645**	**390**	1	5	31	27
MGS-AA, SEK Controls	Multi-ethnic Global	**1745**	**1079**	0	1	12	7
Costa-Rican	Multi-ethnic Global	7	0	**337**	**426**	**54**	**41**
ICCBD-USC	OmniExpress	9	4	2	7	**513**	**1263**
COGS (Caucasian)	PsychArray	19	0	7	4	**2158**	**440**
GPC (Caucasian)	PsychArray	55	9	15	21	**2358**	**1891**
*total N*		**6152**	**3918**	**1234**	**3090**	**6046**	**4534**

Computational phasing was performed for each genotyping batch using Eagle (v2.3.5) [31] and default parameters. Statistical genotype imputation was performed for each genotyping batch using Minimac3 (v2.0.1) [32] and default parameters, using publicly available reference haplotypes from the 1000 Genomes Project (1KGP) Phase 3 [33].

### Relationship inference, population structure and ancestry assignment

We used the KING software package [34] to identify duplicates and infer familial relationships in the full GPC cohort using a set of overlapping, genotyped variants. Within genotyping batches, we excluded from pairs of duplicates the sample with the larger fraction of missing genotypes. Next, we retained one sample from each remaining pair of duplicates or first-degree relatives (i.e. parent−offspring or sibling pairs), preferentially retaining cases from affected/unaffected relative pairs. For diagnostically concordant pairs, we considered the degree (and direction) of case−control imbalance in each of the originating batches in terms effective sample size, where *N*_eff_ = 4/(1/*N*_cases_ + 1/*N*_controls_). We preferentially assigned samples to batches with smaller ratios of *N*_eff_∶*N* when this was ameliorative of case−control imbalance, and updated batch-wise values of *N*_cases_, *N*_controls_ and *N*_eff_ after each assignment.

**Fig. 1:**
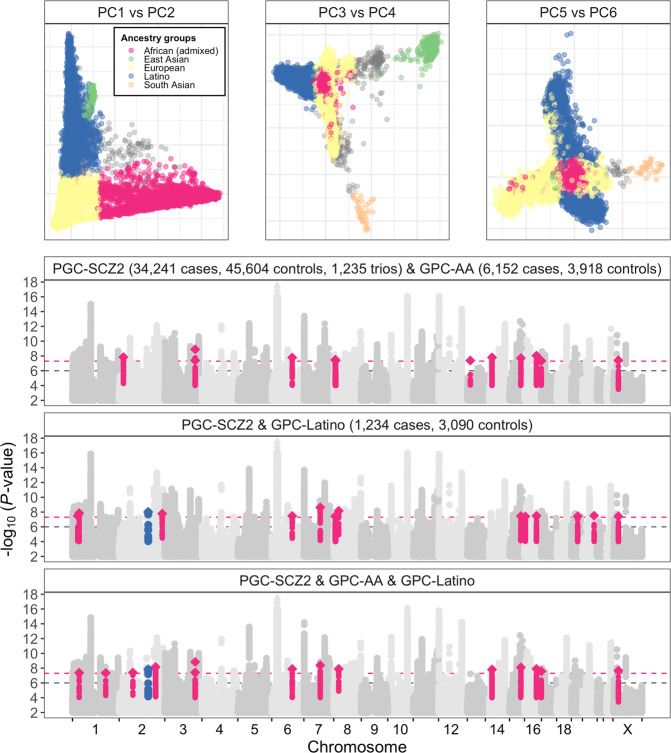
Ancestry assignment and Manhattan plots for trans-ancestry meta-analyses of GPC-AA and GPC-Latino with PGC-SCZ2. **a** PCA-based clustering of GPC participants shaded by broad ancestry assignment. **b** Red and blue dashed lines denote thresholds for genome-wide significance (*P* < 5 × 10^−8^) and replication follow-up in PGC-SCZ2 (*P* < 10^−6^). For newly genome-wide significant regions, the top SNP within a 3 Mb region is displayed as a diamond; nearby SNPs in linkage disequilibrium (*r*^2^ > 0.1) are highlighted

Principal components analysis (PCA) was performed with GCTA (v1.2.4) [35], using a genome-wide genetic relatedness matrix (GRM) estimated for the full GPC dataset and reference samples from the 1KGP Phase 3 data [33] based on 34,918 genotyped SNPs. For each individual, we estimated genome-wide average proportions of African (AFR), European (EUR), Admixed American (AMR), East Asian (EAS), and South Asian (SAS) ancestry from global ancestry PCs using a simple linear mixed model. Using these estimated proportions and defining significant admixture as 25% or more of a given continental origin, we assigned individuals to three broad ancestry groups: 10,070 African (≥25% AFR and <25% AMR, <25% EAS, <25% SAS); 4324 Latino (≥25% AMR and <25% AFR, <25% EAS, <25% SAS); and 10,580 European (<25% AFR, <25% AMR, <25% EAS, <25% SAS) (Fig. 1). Clustering of individuals in each broad ancestry group with the 1KGP reference populations are shown in Supplemental Figs. 1–3. We refer to the admixed African and Latino ancestry GPC cohorts as GPC-AA and GPC-Latino, respectively.

### Genome-wide association and trans-ancestry meta-analysis

Within each broadly defined ancestry group, we tested for association between imputed genotype dosages and a diagnosis of schizophrenia (or SAD) by logistic regression using PLINK [36, 37], and including the first six ancestry PCs and site/cohort indicator variables as covariates. Within each analysis, we retained variants with imputation quality (INFO) of 0.3 or greater and minor allele frequency (MAF) of at least 1%, based on average values calculated for the combined ancestry cohort. We combined association results across ancestry groups under fixed effects (i.e., inverse variance weighted) and Han and Eskin’s random effects (RE2) models, as implemented in METASOFT [38]. The Han and Eskin random effects model is optimized to detect allelic associations in the presence of heterogeneity [38]. We also applied this method to combine male- and female-specific association results for X chromosome variants.

In our primary trans-ancestry meta-analyses, we combine genome-wide summary statistics for African and Latino ancestry GWAS with the PGC-SCZ2 study results. The discovery phase of PGC-SCZ2 included 34,241 cases and 45,604 controls from 46 European and 3 East-Asian case-control studies, and 1235 parent affected offspring trios from 3 family-based samples of European ancestry [12]. The PGC-SCZ2 summary statistics are publicly available (https://www.med.unc.edu/pgc/results-and-downloads) and have been widely utilized in dozens of follow-up studies, and thus represent a meaningful benchmark for genetic analysis. We apply the same filters for SNP association results as described in the original study (INFO ≥ 0.6, MAF ≥ 1%, and present in at least 20 of 49 studies) and interpret the PGC-SCZ2 results as being broadly representative of findings based on European populations.

### Consistency of directions of allelic effects

Linkage disequilibrium (LD) based “clumping” was used to obtain approximately independent sets of SNPs (*r*^2^ < 0.1 within a 500 kilobase (kb) window) using the 1KGP Phase 3 European (EUR) data, and preferentially retaining the most significant SNP in the PGC-SCZ2 analysis (among those meeting filtering criteria in the relevant GPC analysis). For varying *P* value thresholds applied to the PGC-SCZ2 results, we used a binomial sign test to determine if the proportion of same-direction effects in the admixed African or Latino analyses was greater than expected by chance (i.e., a one-sided test of whether this fraction is greater than 0.5). Reciprocal analyses comparing the observed directions of effects in PGC-SCZ2 to the African and Latino ancestry results were also performed, with LD-clumping based on the corresponding 1KGP reference population.

### Polygenic risk score profiling

We performed polygenic risk score profiling based on the PGC-SCZ2 summary statistics (the “training” dataset), testing these scores for association with case−control status in African, Latino, and European cohorts from the GPC (the “target” datasets). For each pair of training and target datasets, results for overlapping SNPs (or indels) meeting quality control requirements (imputation quality ≥ 0.3 and MAF ≥ 1%) were subjected to LD-based clumping in the appropriate reference population from the 1KGP (*r*^2^ < 0.1 within a 500 kb window); for analyses of African, Latino, and European cohorts, we utilized reference data for AFR, AMR, and EUR populations, respectively. For SNPs significant at varying *P* value thresholds (*P*_T_) in the training dataset, individual-level scores were constructed by summing the number of copies of a given allele by its corresponding effect estimate (i.e, the log-transformed odds ratio in the training dataset). We evaluated the significance of case−control differences using logistic regression and covarying ancestry-based principal components (PCs) and a study indicator variable. Predictive values of these scores are reported both in terms of Nagelkerke’s pseudo-*R*^2^ (fmsb package in R) [39] as well as adjusting for sample and population prevalences of 1% for schizophrenia or bipolar disorder (i.e. the liability scale) [40]. We examined how varying strengths of LD among SNPs used to construct a polygenic score influence within- and cross-ancestry genetic prediction by repeating these procedures and increasing the threshold for “clumping” correlated markers (pairwise *r*^2^) to 0.5 and 0.8.

Because genetic prediction is generally worse when comparing training and testing datasets of divergent ancestry, with greater attenuation of predictive value for more divergent populations [27, 28, 41], we constructed analogous polygenic scores based on the African and Latino GWAS results. For within-ancestry prediction, we maintained the independence of training and testing datasets via an iterative “leave-one-out” procedure in which each cohort was omitted, and the remaining samples re-analyzed; the resultant summary statistics represented independent training datasets. For cross-ancestry prediction from the African or Latino GWAS, summary statistics from the primary mega-analysis were utilized.

### Trans-ancestry fine-mapping of schizophrenia loci

We attempted to fine-map 276 autosomal and X-chromosome regions around statistically independent SNPs with association *P* value <10^−6^ in the publicly available PGC-SCZ2 summary statistics. For each index SNP, we considered SNPs correlated at *r*^2^ ≥ 0.6 within a 3 megabase window which had *P* < 10^−4^ in the PGC-SCZ2 discovery analysis. We constructed credible SNP sets by combining their posterior probabilities until the sum exceeded 99%, following the approach of Huang et al. [42]. Credible sets for meta-analytic models representing the PGC-SCZ2 discovery phase and its combined analysis with GPC-AA were compared on the basis of total length and number of credible SNPs, and the smallest observed *P* value among these SNPs; we followed-up regions attaining greater significance in the combined PGC-SCZ2/GPC-AA analysis and for which the credible set in the combined analysis represented a shorter genomic interval than the corresponding interval in the PGC-SCZ2 analysis. We considered a region to be “fine-mapped” if the genomic interval for the reduced credible set was smaller than the corresponding interval for SNPs with LD *r*^2^ ≥ 0.6 to the index SNP (based on 1KGP EUR reference data).

## Results

### Genome-wide association and trans-ancestry meta-analysis

Manhattan and quantile−quantile (QQ) plots for GWAS of admixed African and Latino ancestry individuals are presented in the Supplemental Material (Supplemental Figs. 4 and 5). We calculated the genomic control factor (*λ*) and its value scaled to a sample size of 1000 cases and 1000 controls (*λ*_1000_) from genome-wide distributions of test statistics; these values were 1.04 and 1.008 for the admixed African GWAS, and 1.055 and 1.031 for the Latino GWAS, indicating that our results are not likely to be confounded by population substructure.

Our primary GWAS in admixed African individuals did not yield any SNP findings that reached the accepted threshold for genome-wide significance (*P* < 5 × 10^−8^). In the Latino ancestry GWAS, we identified a novel genome-wide significant association with SNPs in *GALNT13* on chromosome 2q23.3 (rs776877; OR = 1.420, 95% CI:[1.272,1.585]; *P* = 9.62 × 10^−9^) (Supplemental Fig. 6); the associated SNP was not associated in the PGC-SCZ2 analysis (OR = 1.026, 95% CI:[0.994,1.059]; *P* = 0.1215).

Meta-analysis of African ancestry GWAS and PGC-SCZ2 summary statistics yielded 107 independent genome-wide significant SNPs representing 93 physically distinct loci. Of these, 10 were not among the 108 loci reported in the PGC-SCZ2 study (Fig. 1b; Supplemental Table 1).

Combining PGC-SCZ2 and Latino summary statistics, we observed 114 associated SNPs representing 101 loci, 8 of which are newly significant in the current analysis (Fig. 1b; Supplemental Table 2).

Meta-analysis of PGC-SCZ2, African ancestry, and Latino summary statistics revealed two additional significant loci (Fig. 1b; Supplemental Table 3).

### Consistency in directions of allelic effects

Across varying *P* value thresholds in the PGC-SCZ2 dataset and to a high degree of statistical significance overall, the fraction of same-direction effects in the African-ancestry cohort was significantly greater than expected by chance (Supplemental Table 4). We observed a similar pattern of consistency when considering the analysis of African-ancestry individuals and comparing the number of same-direction effects in the PGC-SCZ2 analysis. The observed fraction was significantly greater than expected by chance at more inclusive *P* value thresholds (*P*_T_ < 5 × 10^−4^) accounting for a larger fraction of the genome. This is explainable by the greater degree of statistical enrichment of the PGC-SCZ2 results and corresponding larger number of independent significant findings. The larger number of statistically independent tests genome-wide in the African ancestry GWAS was a reflection of the lower background LD in the African ancestry reference data from the 1KGP. We observed similar results when restricting analyses to individuals with at least 75% African ancestry genome-wide (Supplemental Table 4), and comparable results for comparisons of African and Latino ancestry results (Supplemental Table 5).

### Polygenic risk score profiling

**Fig. 2:**
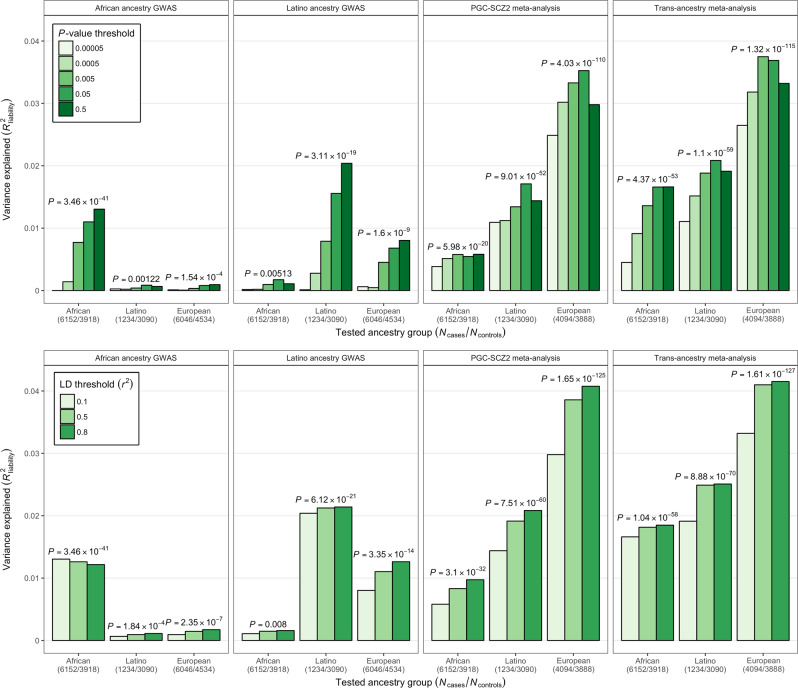
Trans-ancestry association of polygenic risk scores with schizophrenia. For scores based on PGC-SCZ2, GPC-AA or GPC-Latino, and meta-analysis results, the variance in risk explained in the other study is shown on the *y*-axis in terms of *R*^2^ on the liability scale. **a** Scores based on various *P* value inclusion thresholds are displayed as shaded bars; **b** scores based on *P*_T_ < 0.5 and varying pairwise LD between SNPs are displayed as shaded bars. Analyses of PGC-SCZ2 and meta-analysis scores utilized an independent cohort of European ancestry GPC participants

Consistent with previous reports demonstrating the generalizability of polygenic findings for schizophrenia across diverse populations [14, 43, 44], individual-level scores constructed from PGC-SCZ2 summary statistics were significantly associated with case−control status in admixed African, Latino, and European cohorts in the current study (Fig. 2a). When considering scores constructed from approximately independent common variants (pairwise *r*^*2*^ < 0.1), we observed the best overall prediction at a *P* value threshold (*P*_T_) of 0.05; these scores explained ~3.5% of the variance in schizophrenia liability among Europeans (*P* = 4.03 × 10^−110^), ~1.7% among Latino individuals (*P* = 9.02 × 10^−52^), and ~0.5% among admixed African individuals (*P* = 8.25 × 10^−19^) (Fig. 2a; Supplemental Table 6). Consistent with expectation, when comparing results for scores constructed from larger numbers of nonindependent SNPs, we generally observed an improvement in predictive value (Fig. 2b; Supplemental Table 7).

Polygenic scores based on African ancestry GWAS results were significantly associated with schizophrenia among admixed African individuals, attaining the best overall predictive value when constructed from approximately independent common variants (pairwise *r*^*2*^ < 0.1) with *P*_T_ ≤ 0.5 in the discovery analysis (Fig. 2a and Supplemental Table 6); this score explained ~1.3% of the variance in schizophrenia liability (*P* = 3.47 × 10^−41^). Scores trained on African ancestry GWAS results also significantly predicted case−control status across populations; scores based on a *P*_T_ ≤ 0.5 and pairwise *r*^*2*^ < 0.8 explained ~0.2% of the variability in liability in Europeans (*P* = 2.35 × 10^−7^) and ~0.1% among Latino individuals (*P* = 0.000184) (Fig. 2b and Supplemental Table 7). Similarly, scores constructed from Latino GWAS results (*P*_T_ *≤* 0.5) were of greatest predictive value among Latinos (liability *R*^*2*^ = 2%; *P* = 3.11 × 10^−19^) and Europeans (liability *R*^*2*^ = 0.8%; *P* = 1.60 × 10^−9^); with scores based on *P*_T_ *≤* 0.05 and pairwise *r*^2^ < 0.1 showing nominally significant association with case-control status among African ancestry individuals (liability *R*^*2*^ = 0.2%; *P* = 0.00513).

We next considered polygenic scores constructed from trans-ancestry meta-analysis of PGC-SCZ2 summary statistics and our African and Latino GWAS, which revealed increased significance and improved predictive value in all three ancestries. Among African ancestry individuals, meta-analytic scores based on *P*_T_ *≤* 0.5 explained ~1.7% of the variance (*P* = 4.37 × 10^−53^); while scores based on *P*_T_ *≤* 0.05 accounted for ~2.1% and ~3.7% of the variability in liability among Latino (*P* = 1.10 × 10^−59^) and European individuals (*P* = 1.73 × 10^−114^), respectively.

We then considered a “baseline” generalized linear model including the PGC-SCZ2 score and covariates as predictors and compared this to a joint model incorporating African- and/or Latino-trained scores by a log-likelihood ratio test. Consistent with our observation that polygenic scores constructed from trans-ancestry meta-analysis results yielded improved prediction at genome-wide *P*_T_, joint models incorporating both PGC-SCZ2 and ancestry-specific scores yielded significant improvements in goodness-of-fit (Supplemental Table 8).

We also considered whether these schizophrenia polygenic risk scores also indexed risk of bipolar disorder in independent cases from the GPC. We observed a similar pattern of findings as those reported above, albeit with systematic attenuation of signal in terms of explained variance and statistical significance, which is expected (Supplemental Table 9). Critically, scores constructed from PGC-SCZ2 and trans-ancestry meta-analysis results were significantly associated with a diagnosis of bipolar disorder in African, Latino, and European populations (*P* < 10^−5^).

### Trans-ancestry fine-mapping of schizophrenia loci

We next sought to evaluate the extent to which combining PGC-SCZ2 summary statistics with GPC-AA and GPC-Latino results would yield improved fine-mapping resolution at implicated loci. For replicated associations from PGC-SCZ2 that increased in significance following trans-ancestry meta-analysis, we compared “credible sets” of SNPs constructed from trans-ancestry meta-analysis summary statistics to those based on the PGC-SCZ2 results alone. We interpreted any reductions in both the number of SNPs comprising the 99% credible set and the length of the corresponding genomic interval as evidence of improved fine-mapping resolution.

Among 128 statistically significant associations in the PGC-SCZ2 study, we successfully fine-mapped 12 regions by trans-ancestry meta-analysis with African ancestry GWAS summary statistics (Table 2). Meta-analysis of PGC-SCZ2 and Latino summary statistics yielded reductions in the credible set for nine regions (Supplemental Table 10), including one newly significant region. Combining PGC-SCZ2, African ancestry, and Latino summary statistics showed improved fine-mapping resolution for two additional PGC-SCZ2 regions (rs6670165 and chr11_46350213_D); and for one of two2 regions that saw credible set reductions from meta-analysis with either African or Latino ancestry results, this improved fine-mapping resolution was further enhanced in the combined analysis (Supplemental Table 11).

**Table 2:** Improved fine-mapping resolution at 12 established schizophrenia loci by trans-ancestry meta-analysis of PGC-SCZ2 and GPC-AA.

Index SNP	Chr	99% credible set: PGC-SCZ2			99% credible set: Meta-analysis			99% credible set: reduction	
		Location (GRCh37)	SNPs	Interval (kb)	Location (GRCh37)	SNPs	Interval (kb)	SNPs	Interval (kb)
rs12129573	1	73,766,431–73,988,149	162	221.72	73,768,366–73,988,149	154	219.78	8	1.94
rs6670165	1	177,247,854–177,300,809	22	52.96	177,247,854–177,280,121	8	32.27	14	20.69
chr2_146436222_I	2	146,419,047–146,441,828	22	22.78	146,419,170–146,440,672	18	21.5	4	1.28
rs2909457	2	162,798,581–162,891,848	28	93.27	162,798,581–162,856,148	27	57.57	1	35.7
rs6466055	7	104,597,669–105,063,372	111	465.7	104,598,479–105,058,488	104	460.01	7	5.69
chr11_46350213_D	11	46,343,189–46,684,677	98	341.49	46,373,311–46,673,344	56	300.03	42	41.46
rs679087	12	29,905,251–29,939,628	13	34.38	29,905,251–29,934,586	12	29.34	1	5.04
rs12903146	15	61,831,680–61,887,768	36	56.09	61,831,863–61,873,251	28	41.39	8	14.7
rs78322266	18	52,987,161–53,173,173	49	186.01	52,987,161–53,105,738	30	118.58	19	67.44
rs72934570	18	53,533,189–53,585,664	17	52.48	53,533,189–53,584,013	15	50.82	2	1.65
rs56873913	19	50,067,508–50,103,252	34	35.74	50,078,276–50,103,252	27	24.98	7	10.77
rs9607782	22	41,418,154–41,627,775	18	209.62	41,587,556–41,587,556	1	0	17	209.62

For each index SNP, descriptives of 99% credible sets constructed from PGC-SCZ2 and meta-analysis results are displayed; credible sets are summarized in terms of genomic coordinates, number of SNPs, and length of the genomic interval in kilobases (kb), and improvement in fine-mapping resolution is given in terms of reductions in the number of SNPs and corresponding interval length

The degree of improved fine-mapping resolution varied between loci, and in two instances was reduced to a single SNP (rs9607782 and rs211829 in Table 2 and Supplemental Table 10, respectively). Importantly, for these fine-mapped regions, genomic intervals corresponding to 99% credible set were smaller than corresponding intervals defined by SNPs with LD *r*^*2*^ > 0.6 with the index SNP (based on 1KGP EUR reference data).

**Fig. 3:**
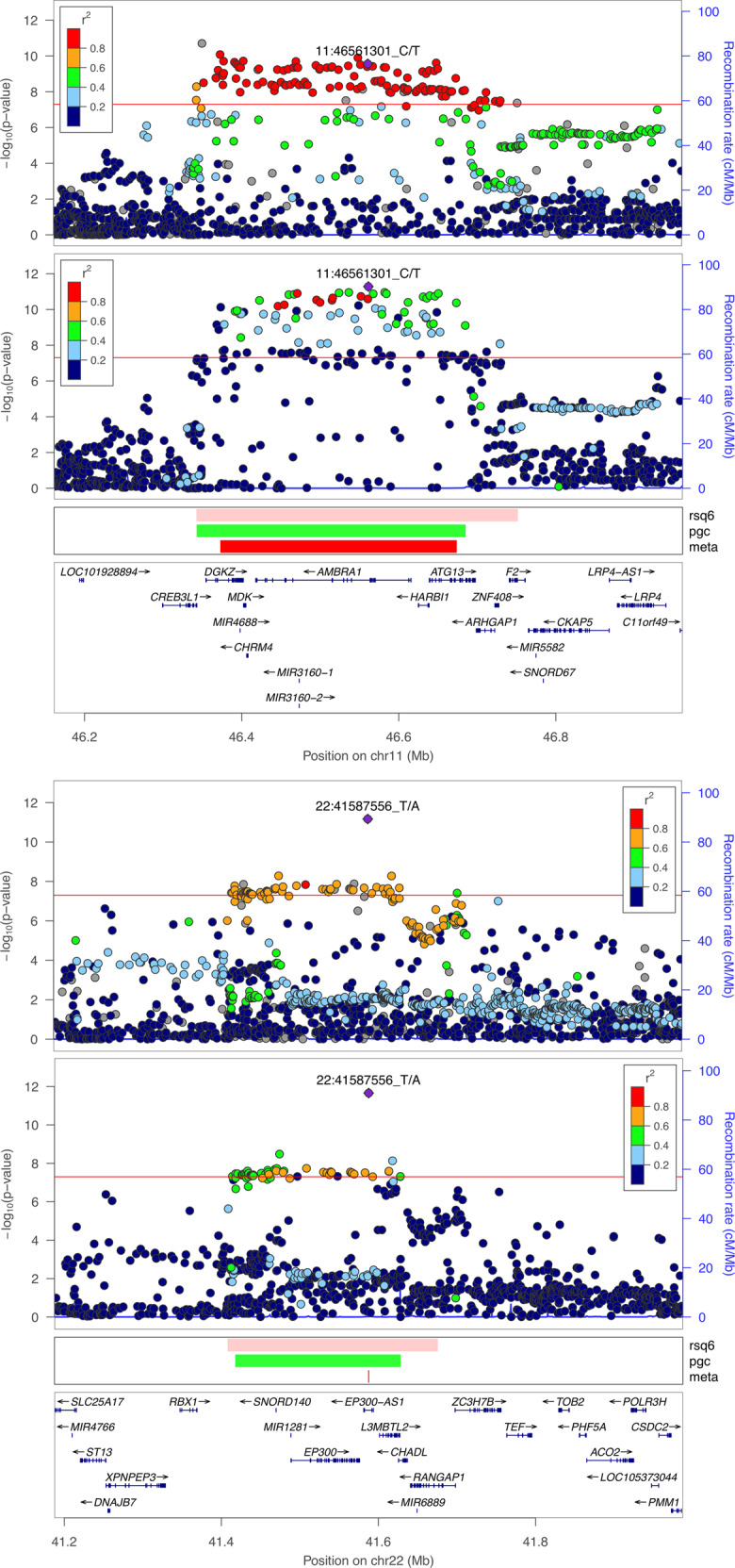
Regional association plots for selected schizophrenia associations with improved fine-mapping resolution in trans-ancestry meta-analysis. For each selected region, association results for PGC-SCZ2 and meta-analysis of PGC-SCZ2 with GPC-AA are shown in the first and second panels, respectively. The strength of LD of each SNP with the “index” SNP, displayed as a large purple diamond, is indicated by its color. Genomic intervals corresponding to SNPs with LD *r*^2^ > 0.6 to the index SNP (“rsq6”) and 99% credible sets in PGC-SCZ2 (“pgc”) and the present analysis (“meta”) are displayed. Plots were created using the LocusZoom standalone software [54]

For selected regions on chromosomes 11p11.2 and 22q13.2, Fig. 3 displays regional association results for PGC-SCZ2 and trans-ancestry meta-analysis of PGC-SCZ2 and GPC-AA.

## Discussion

We have undertaken the largest genetic association study of schizophrenia in persons of African ancestry to date and provide important benchmarks for the generalizability of aggregate findings across diverse populations. We observed a significant excess of SNPs with consistent directions of allelic effect across studies and populations as well as robust enrichments of identified risk alleles among cases compared to controls. We demonstrate that combining European and African ancestry data has the potential to generate empirical support for specific genetic variants, and to refine implicated risk loci by trans-ancestry fine-mapping. Critically, aggregate polygenic risk scores derived from the largest published GWAS of SCZ to date have markedly attenuated predictive value among non-Europeans, presenting an imperative for increased diversity of participants in psychiatric genetics research.

Among admixed African cases and controls, we were able to explain a larger fraction of variance using polygenic scores constructed from our African ancestry GWAS results, and European and Latino cases were found to carry more of the African-derived score alleles than ancestry-matched controls. The predictive value of PGC-SCZ2 scores was comparable for European and Latino cohorts, but considerably attenuated in admixed African ancestry individuals. Importantly, meta-analysis of PGC-SCZ2 and African ancestry GWAS results yielded the best “training” dataset overall, with resultant scores explaining more variance among European and African ancestry individuals than corresponding scores based on either ancestry alone. Recalling the seminal findings of the International Schizophrenia Consortium (2009), polygenic scores based on a larger European cohort showed attenuated effects in the MGS-AA sample [14], reflecting aggregate differences in allele frequencies and patterns of linkage disequilibrium. Consistent with expectation, the overall predictive value of these scores was improved, both within- and across-ancestries, by achieving more complete coverage of the genome through use of a larger set of variants. Taken together, these results highlight that the utility of polygenic scoring methodologies—in both basic research and in terms of potential clinical applications—relies on the availability of appropriately matched “training” and “testing” samples. Larger and more inclusive GWAS are necessary to ensure that advances in genomic medicine, including improved risk prediction, benefit the entirety of humanity.

While GWAS of schizophrenia in admixed African ancestry individuals did not yield any genome-wide significant findings, our analysis of Latino cases and controls revealed a novel genome-wide association with SNPs in *GALNT13* at 2q23.3. This locus encodes polypeptide *N*-acetylgalactosaminyltransferase 13, which has been shown to be specifically expressed in neurons and may be responsible for synthesizing Tn antigen; the 3′ UTR region contains two microRNA target sequences. The associated allele at the leading SNP at this locus (rs776877) has an odds ratio of ~1.4, which is larger than expected given that the majority of associated variants in PGC-SCZ2 have an odds ratio less than 1.2. This may be attributable to the phenomenon described as “winner’s curse” [45]. This SNP yielded significance evidence of heterogeneity of effect sizes in the trans-ancestry analysis of PGC-SCZ2, GPC-AA and GPC-Latino (Cochran’s *Q* = 33.1605; *P* = 6.30 × 10^–8^; I^2^ = 93.97%). It is also worth noting that a prior study of psychosis in Mexican and Central American families yielded some evidence of linkage to 2q33 [46]. However, confirmation of *GALNT13* as a schizophrenia risk locus will require detailed follow-up and replication in an independent Latino cohort.

Meta-analysis of African ancestry results with PGC-SCZ2 summary statistics yielded 94 associated loci, of which 11 were not among the 108 previously reported, and 7 were newly genome-wide significant. These additional loci were significant at *P* < 10^−6^ in the PGC-SCZ2 discovery phase but did not attain genome-wide significance in a combined analysis with 1513 cases and 66,236 controls from deCODE genetics. It is noteworthy that the effective sample size (*N*_eff_) of the deCODE replication sample—the total sample size adjusted for imbalanced numbers of cases and controls—is smaller than the effective sample size of the African ancestry cohort (*N*_eff_ = 5 917 vs. *N*_eff_ = 9 574). That we observed fewer genome-wide significant associations overall is consistent with expectation that gains in statistical power from increasing sample size will be largest when adding ancestry-matched subjects. With greater “genetic distance” (e.g. fixation index, or *F*_ST_) between the discovery and replication samples, we would expect greater attenuation in terms of realized gains in statistical power relative to increase in sample size. For example, consider that meta-analysis with Latino results (*N*_eff_ = 3 527) yielded 101 loci, including 12 newly replicated loci.

Comparing the 18 newly significant loci reported here (Supplemental Tables 1–3) to findings from a recently published meta-analysis of PGC-SCZ2 and CLOZUK2 [16], we observe just six overlapping genome-wide significant loci. Noting the large sample size of the CLOZUK2 sample (5220 cases and 18,823 controls not included in the PGC-SCZ2 analysis), this argues that trans-ancestry meta-analysis has the potential to enlarge the scope of GWAS findings and lead to identification of novel associations.

Fine-mapping approaches often utilize functional annotations (e.g. predicted deleteriousness of nonsynonymous variants) to identify a likely causal variant at an associated locus [47, 48], and methods that leverage population differences in patterns of linkage disequilibrium have also been described [49, 50]. Our approach was to compare credible SNP sets constructed from PGC-SCZ2 and our trans-ancestry meta-analysis results, interpreting a reduction in the number of credible SNPs and length of the corresponding genomic interval as indication of improved fine-mapping resolution. Among 128 associated SNPs identified in the PGC-SCZ2 analysis, 41 increased in significance in the African ancestry meta-analysis; for 12 of these regions, we observed a concomitant reduction in the number of SNPs comprising the credible set. It can be expected that larger sample sizes will yield larger numbers of fine-mapped loci and enhanced fine-mapping resolution.

### Limitations

We do not specifically model admixture within individuals or adjust for local ancestry in tests of common variant association, instead adjusting for global ancestry proportions within broadly defined ancestry groups. Importantly, our resultant test-statistic distributions do not suggest significant confounding by population substructure. It is likely that much larger samples of African and Latino ancestry are needed to capture the extensive genetic diversity present in these populations [51, 52].

We do not give specific consideration to enrichment of observed associations in particular biological pathways or other functional annotations (e.g. tissue-specific eQTLs), or evidence of cross-ancestry and cross-trait genetic correlations. This is in part owing to our concern that many current and trending methods utilize reference LD information, and the suitability of these data to admixed populations is an unresolved, empirical question.

### Conclusions

We have conducted the largest GWAS of schizophrenia among admixed African individuals to date and demonstrate the potential of more diverse studies to refine the catalog of schizophrenia risk loci and enhance the generalizability of aggregate genetic findings. Addressing disparities in representation of African and Latino ancestries in psychiatric genetics research presents both scientific opportunities and imperatives [53], necessitating greater community engagement and genotyping initiatives at population-scale.

## Supplementary information

Supplemental Materials

## References

[1] Sullivan PF, Kendler KS, Neale MC (2003). Schizophrenia as a complex trait: evidence from a meta-analysis of twin studies. Arch Gen Psychiatry.

[2] Bergen SE, O’Dushlaine CT, Ripke S, Lee PH, Ruderfer DM, Akterin S (2012). Genome-wide association study in a Swedish population yields support for greater CNV and MHC involvement in schizophrenia compared with bipolar disorder. Mol Psychiatry.

[3] Karayiorgou M, Morris MA, Morrow B, Shprintzen RJ, Goldberg R, Borrow J (1995). Schizophrenia susceptibility associated with interstitial deletions of chromosome 22q11. Proc Natl Acad Sci USA.

[4] International Schizophrenia Consortium. (2008). Rare chromosomal deletions and duplications increase risk of schizophrenia. Nature.

[5] Stefansson H, Rujescu D, Cichon S, Pietiläinen OPH, Ingason A, Steinberg S (2008). Large recurrent microdeletions associated with schizophrenia. Nature.

[6] McCarthy SE, Makarov V, Kirov G, Addington AM, McClellan J, Yoon S (2009). Microduplications of 16p11.2 are associated with schizophrenia. Nat Genet.

[7] Purcell SM, Moran JL, Fromer M, Ruderfer D, Solovieff N, Roussos P (2014). A polygenic burden of rare disruptive mutations in schizophrenia. Nature.

[8] Fromer M, Pocklington AJ, Kavanagh DH, Williams HJ, Dwyer S, Gormley P (2014). De novo mutations in schizophrenia implicate synaptic networks. Nature.

[9] Nguyen HT, Bryois J, Kim A, Dobbyn A, Huckins LM, Munoz-Manchado AB (2017). Integrated Bayesian analysis of rare exonic variants to identify risk genes for schizophrenia and neurodevelopmental disorders. Genome Med.

[10] Marshall CR, Howrigan DP, Merico D, Thiruvahindrapuram B, Wu W, Greer DS (2017). Contribution of copy number variants to schizophrenia from a genome-wide study of 41,321 subjects. Nat Genet.

[11] Genovese G, Fromer M, Stahl EA, Ruderfer DM, Chambert K, Landén M (2016). Increased burden of ultra-rare protein-altering variants among 4,877 individuals with schizophrenia. Nat Neurosci.

[12] Schizophrenia Working Group of the Psychiatric Genomics, Consortium. (2014). Biological insights from 108 schizophrenia-associated genetic loci. Nature.

[13] Lee SH, DeCandia TR, Ripke S, Yang J, Schizophrenia Psychiatric Genome-WideAssociation Study Consortium (PGC-SCZ), International Schizophrenia Consortium (ISC) (2012). Estimating the proportion of variation in susceptibility to schizophrenia captured by common SNPs. Nat Genet.

[14] Purcell SM, Wray NR, Stone JL, Visscher PM, O’Donovan MC, International Schizophrenia, Consortium (2009). Common polygenic variation contributes to risk of schizophrenia and bipolar disorder. Nature.

[15] Shi H, Kichaev G, Pasaniuc B (2016). Contrasting the genetic architecture of 30 complex traits from summary association data. Am J Hum Genet.

[16] Pardiñas AF, Holmans P, Pocklington AJ, Escott-Price V, Ripke S, Carrera N (2018). Common schizophrenia alleles are enriched in mutation-intolerant genes and in regions under strong background selection. Nat Genet.

[17] Sullivan PF, Agrawal A, Bulik CM, Andreassen OA, Børglum AD, Breen G (2018). Psychiatric genomics: an update and an agenda. Am J Psychiatry.

[18] Schizophrenia Psychiatric Genome-Wide Association Study, Consortium. (2011). Genome-wide association study identifies five new schizophrenia loci. Nat Genet.

[19] Ripke S, O’Dushlaine C, Chambert K, Moran JL, Kähler AK, Akterin S (2013). Genome-wide association analysis identifies 13 new risk loci for schizophrenia. Nat Genet.

[20] Need AC, Goldstein DB (2009). Next generation disparities in human genomics: concerns and remedies. Trends Genet.

[21] Popejoy AB, Fullerton SM (2016). Genomics is failing on diversity. Nature.

[22] de Candia TR, Lee SH, Yang J, Browning BL, Gejman PV, Levinson DF (2013). Additive genetic variation in schizophrenia risk is shared by populations of African and European descent. Am J Hum Genet.

[23] Li Z, Chen J, Yu H, He L, Xu Y, Zhang D (2017). Genome-wide association analysis identifies 30 new susceptibility loci for schizophrenia. Nat Genet.

[24] Shi J, Levinson DF, Duan J, Sanders AR, Zheng Y, Pe’er I (2009). Common variants on chromosome 6p22.1 are associated with schizophrenia. Nature.

[25] Stefansson H, Ophoff RA, Steinberg S, Andreassen OA, Cichon S, Rujescu D (2009). Common variants conferring risk of schizophrenia. Nature.

[26] consortium, Converge. (2015). Sparse whole-genome sequencing identifies two loci for major depressive disorder. Nature.

[27] Martin AR, Gignoux CR, Walters RK, Wojcik GL, Neale BM, Gravel S (2017). Human demographic history impacts genetic risk prediction across diverse populations. Am J Hum Genet.

[28] Duncan L, Shen H, Gelaye B, Ressler K, Feldman M, Peterson R, et al. Analysis of polygenic score usage and performance in diverse human populations [Internet]. *bioRxiv*. 2018 [cited 2019]; 398396. Available from: https://www.biorxiv.org/content/early/2018/11/03/398396. 10.1038/s41467-019-11112-0 PMC6658471 31346163

[29] Barrett JC, Cardon LR (2006). Evaluating coverage of genome-wide association studies. Nat Genet.

[30] Marchini J, Cardon LR, Phillips MS, Donnelly P (2004). The effects of human population structure on large genetic association studies. Nat Genet.

[31] Loh P-R, Danecek P, Palamara PF, Fuchsberger C, A Reshef Y, K Finucane H (2016). Reference-based phasing using the Haplotype Reference Consortium panel. Nat Genet.

[32] Das S, Forer L, Schönherr S, Sidore C, Locke AE, Kwong A (2016). Next-generation genotype imputation service and methods. Nat Genet.

[33] Auton A, Brooks LD, Durbin RM, Garrison EP, Kang HM, 1000 Genomes Project Consortium (2015). A global reference for human genetic variation. Nature.

[34] Manichaikul A, Mychaleckyj JC, Rich SS, Daly K, Sale M, Chen W-M (2010). Robust relationship inference in genome-wide association studies. Bioinformatics.

[35] Yang J, Lee SH, Goddard ME, Visscher PM (2011). GCTA: a tool for genome-wide complex trait analysis. Am J Hum Genet.

[36] Chang CC, Chow CC, Tellier LC, Vattikuti S, Purcell SM, Lee JJ (2015). Second-generation PLINK: rising to the challenge of larger and richer datasets. Gigascience.

[37] Purcell S, Neale B, Todd-Brown K, Thomas L, Ferreira MAR, Bender D (2007). PLINK: a tool set for whole-genome association and population-based linkage analyses. Am J Hum Genet.

[38] Han B, Eskin E (2011). Random-effects model aimed at discovering associations in meta-analysis of genome-wide association studies. Am J Hum Genet.

[39] Nakazawa M (2007). Practices of medical and health data analysis using R..

[40] Lee SH, Goddard ME, Wray NR, Visscher PM (2012). A better coefficient of determination for genetic profile analysis. Genet Epidemiol.

[41] Scutari M, Mackay I, Balding D (2016). Using genetic distance to infer the accuracy of genomic prediction. PLoS Genet.

[42] Huang H, Fang M, Jostins L, Umićević Mirkov M, Boucher G, Anderson CA (2017). Fine-mapping inflammatory bowel disease loci to single-variant resolution. Nature.

[43] Ikeda M, Aleksic B, Kinoshita Y, Okochi T, Kawashima K, Kushima I (2011). Genome-wide association study of schizophrenia in a Japanese population. Biol Psychiatry.

[44] Yue W-H, Wang H-F, Sun L-D, Tang F-L, Liu Z-H, Zhang H-X (2011). Genome-wide association study identifies a susceptibility locus for schizophrenia in Han Chinese at 11p11. 2. Nat Genet.

[45] Zollner S, Pritchard JK (2007). Overcoming the winner’s curse: estimating penetrance parameters from case-control data. Am J Hum Genet.

[46] Escamilla M, Hare E, Dassori AM, Peralta JM, Ontiveros A, Nicolini H (2009). A schizophrenia gene locus on chromosome 17q21 in a new set of families of Mexican and central american ancestry: evidence from the NIMH Genetics of schizophrenia in latino populations study. Am J Psychiatry.

[47] Kichaev G, Roytman M, Johnson R, Eskin E, Lindström S, Kraft P (2017). Improved methods for multi-trait fine mapping of pleiotropic risk loci. Bioinformatics.

[48] Hormozdiari F, Kostem E, Kang EY, Pasaniuc B, Eskin E (2014). Identifying causal variants at loci with multiple signals of association. Genetics.

[49] Morris AP (2011). Transethnic meta-analysis of genomewide association studies. Genet Epidemiol.

[50] Mägi R, Horikoshi M, Sofer T, Mahajan A, Kitajima H, Franceschini N (2017). Trans-ethnic meta-regression of genome-wide association studies accounting for ancestry increases power for discovery and improves fine-mapping resolution. Hum Mol Genet.

[51] Mathias RA, Taub MA, Gignoux CR, Fu W, Musharoff S, O’Connor TD (2016). A continuum of admixture in the Western Hemisphere revealed by the African Diaspora genome. Nat Commun.

[52] Wang S, Ray N, Rojas W, Parra MV, Bedoya G, Gallo C (2008). Geographic patterns of genome admixture in Latin American Mestizos. PLoS Genet.

[53] Forero DA, Vélez-van-Meerbeke A, Deshpande SN, Nicolini H, Perry G (2014). Neuropsychiatric genetics in developing countries: Current challenges. World J Psychiatry.

[54] Pruim RJ, Welch RP, Sanna S, Teslovich TM, Chines PS, Gliedt TP (2010). LocusZoom: regional visualization of genome-wide association scan results. Bioinformatics.

