# Supplementary material for: Contributions of common genetic variants to risk of schizophrenia among individuals of African and Latino ancestry
Source: Mol Psychiatry. 2019 Oct 7;25(10):2455–67. doi: 10.1038/s41380-019-0517-y (PMC7515843; doi:10.1038/s41380-019-0517-y)
Supplement: Supplementary file 1 — Supplemental Materials [file 41380_2019_517_MOESM1_ESM.docx]

# **Supplemental Materials**

## **Ascertainment and diagnosis**

GPC participants were drawn from cases and controls living and being treated in local communities and healthcare delivery systems. All participants enrolled as probable cases were interviewed using the Diagnostic Interview for Psychosis and Affective Disorders (DI-PAD), a semi-structured clinical interview administered by mental health professionals. The DI-PAD was developed specifically for the GPC study using the same principles as were applied in the development of the Diagnostic Interview for Psychosis – Diagnostic Module (DIP-DM) [(1)](https://paperpile.com/c/pLC4Y0/ou9H), and incorporates questions developed for the Diagnostic Interview for Genetic Studies (DIGS) [(2)](https://paperpile.com/c/pLC4Y0/8l39). Inclusion criteria for cases include meeting lifetime diagnostic criteria for schizophrenia or schizoaffective disorder (any subtype) in accordance with the OPCRIT algorithms for DSM-IV [(3)](https://paperpile.com/c/pLC4Y0/aaAu) and/or ICD-10 [(4)](https://paperpile.com/c/pLC4Y0/EfBt) criteria, and/or DSM-5 [(5)](https://paperpile.com/c/pLC4Y0/L7oK). Individuals reporting no lifetime symptoms indicative of psychosis or mania and who have no first-degree relatives with these symptoms are included as control participants. Exclusion criteria included any premorbid organic mental disorders (i.e., epilepsy, CNS infection, significant head trauma, mental retardation), and premorbid history of significant drug or alcohol dependence by DSM IV/5 that confounds the diagnosis of schizophrenia. All participants gave written informed consent and the IRB of the participating institutions approved the protocol.

Blood-derived DNA for additional cases and controls meeting the same inclusion and exclusion criteria were obtained from the NIMH Repository, including the Molecular Genetics of Schizophrenia (MGS) [(6–8)](https://paperpile.com/c/pLC4Y0/kQBa+4nKV+P0A0), COGS [(9)](https://paperpile.com/c/pLC4Y0/FzED), and PAARTNERS [(10)](https://paperpile.com/c/pLC4Y0/Tve3) studies.

##

## **Supplemental Tables**

### **Supplemental Table 1. Newly genome-wide significant findings in the meta-analysis of PGC-SCZ2 and GPC-AA.** For each associated SNP, *Variant* indicates chromosome, genomic coordinates (GRCh37), reference and alternative allele, and *RSID* is its dbSNP identifier; for a given analysis, *Freq* is the frequency of the reference allele, OR and SE are the odds ratio and its standard error, and *P* is its significance; *P*_meta_ is the meta-analysis *P*-value; and *P_het_* is the significance of Cochran’s test for heterogeneity.

| Variant | RSID | PGC-SCZ2 | | | |  | GPC-AA | | | | *P*_meta_ | *P_het_* |
| --- | --- | --- | --- | --- | --- | --- | --- | --- | --- | --- | --- | --- |
|  |  | Freq | INFO | OR (SE) | *P* |  | Freq | INFO | OR (SE) | *P* |  |  |
| 13:35194022:C:T | rs1329313 | 0.450 | 0.981 | 0.947 (0.011) | 3.41×10^-7^ |  | 0.604 | 0.976 | 0.937 (0.030) | 0.0286 | **4.14×10^-8^** | 0.727 |
| 14:35594106:C:G | rs2415261 | 0.836 | 0.990 | 1.074 (0.014) | 2.85×10^-7^ |  | 0.738 | 0.981 | 1.092 (0.035) | 0.0116 | **1.58×10^-8^** | 0.661 |
| 15:83254708:A:G | rs783540 | 0.582 | 1.020 | 0.944 (0.011) | 5.86×10^-8^ |  | 0.561 | 1.004 | 0.950 (0.030) | 0.0791 | **1.98×10^-8^** | 0.849 |
| 16:89609418:T:G | rs34753377 | 0.644 | 0.862 | 1.066 (0.012) | 1.06×10^-7^ |  | 0.644 | 0.875 | 1.050 (0.033) | 0.134 | **4.61×10^-8^** | 0.662 |
| 2:22754466:A:G | rs1509378 | 0.350 | 0.920 | 1.064 (0.012) | 8.37×10^-8^ |  | 0.376 | 0.922 | 1.065 (0.031) | 0.0453 | **1.44×10^-8^** | 0.974 |
| 3:161779956:T:C | rs28730912 | 0.270 | 0.746 | 1.069 (0.012) | 5.09×10^-8^ |  | 0.433 | 0.808 | 1.101 (0.033) | 0.0033 | **1.30×10^-9^** | 0.403 |
| 8:10032894:C:A | rs11993663 | 0.692 | 0.995 | 0.943 (0.011) | 1.46×10^-7^ |  | 0.641 | 1.000 | 0.950 (0.031) | 0.0913 | **4.21×10^-8^** | 0.840 |
| X:29276212:A:C | rs5943630 | 0.330 | 1.450 | 1.052 (0.009) | 6.36×10^-8^ |  | 0.528 | 1.205 | 1.035 (0.033) | 0.171 | **3.74×10^-8^** | 0.450 |

**Supplemental Table 2. Newly genome-wide significant findings in the meta-analysis of PGC-SCZ2 and GPC-Latino.** Abbreviations are described in Supplemental Table 1.

| Variant | RSID | PGC-SCZ2 | | | |  | GPC-Latino | | | | *P*_meta_ | *P_het_* |
| --- | --- | --- | --- | --- | --- | --- | --- | --- | --- | --- | --- | --- |
|  |  | Freq | INFO | OR (SE) | *P* |  | Freq | INFO | OR (SE) | *P* |  |  |
| 1:29141155:G:A | rs533123 | 0.181 | 0.968 | 0.928 (0.014) | 1.97×10^-7^ |  | 0.160 | 0.973 | 0.842 (0.072) | 0.0178 | **3.36×10^-8^** | 0.188 |
| 1:36616941:T:G | rs7537052 | 0.336 | 0.970 | 0.943 (0.012) | 3.31×10^-7^ |  | 0.506 | 0.971 | 0.837 (0.051) | 0.0005 | **1.40×10^-8^** | 0.0223 |
| 16:4497537:G:C | rs6500603 | 0.314 | 0.993 | 1.062 (0.012) | 2.80×10^-7^ |  | 0.395 | 0.992 | 1.126 (0.051) | 0.0200 | **3.95×10^-8^** | 0.261 |
| 19:33897934:T:G | rs10425465 | 0.735 | 0.957 | 1.072 (0.013) | 8.27×10^-8^ |  | 0.644 | 0.973 | 1.078 (0.053) | 0.154 | **3.79×10^-8^** | 0.907 |
| 2:154797530:A:C | rs776877 | 0.879 | 0.941 | 1.026 (0.016) | 0.122 |  | 0.705 | 0.984 | 1.420 (0.056) | 2.70×10^-10^ | **9.62×10^-9^** | 1.93×10^-8^ |
| 2:229288849:C:T | rs140987748 | 0.480 | 0.789 | 0.937 (0.012) | 8.84×10^-8^ |  | 0.375 | 0.970 | 0.895 (0.052) | 0.0317 | **1.66×10^-8^** | 0.383 |
| 7:87244960:C:T | rs13233308 | 0.534 | 0.966 | 1.055 (0.011) | 5.70×10^-7^ |  | 0.625 | 0.984 | 1.245 (0.051) | 1.79×10^-5^ | **2.37×10^-9^** | 0.00150 |
| 8:26272768:G:A | rs73219805 | 0.835 | 0.980 | 0.931 (0.014) | 2.99×10^-7^ |  | 0.841 | 1.008 | 0.782 (0.065) | 0.000159 | **6.90×10^-9^** | 0.00894 |

###

###

### **Supplemental Table 3. Newly genome-wide significant findings in the meta-analysis of PGC-SCZ2, GPC-AA, and GPC-Latino.** Abbreviations are described in Supplemental Table 1.

| Variant | RSID | PGC-SCZ2 | | | |  | GPC-AA | | | |  | GPC-Latino | | | | *P*_meta_ | *P_het_* |
| --- | --- | --- | --- | --- | --- | --- | --- | --- | --- | --- | --- | --- | --- | --- | --- | --- | --- |
|  |  | Freq | INFO | OR (SE) | *P* |  | Freq | INFO | OR (SE) | *P* |  | Freq | INFO | OR (SE) | *P* |  |  |
| 1:177766021:C:G | rs75608493 | 0.880 | 0.915 | 1.093 (0.018) | 3.38×10^-7^ |  | 0.993 | 0.883 | 1.140 (0.100) | 0.191 |  | 0.912 | 0.929 | 1.188 (0.089) | 0.0535 | **4.53×10^-8^** | 0.612 |
| 2:73161551:A:G | rs2077586 | 0.728 | 0.915 | 1.067 (0.012) | 1.53×10^-7^ |  | 0.759 | 0.914 | 1.048 (0.034) | 0.169 |  | 0.599 | 0.963 | 1.067 (0.053) | 0.220 | **3.97×10^-8^** | 0.881 |

###

### **Supplemental Table 4.** Binomial sign test for consistency of directions of allelic effect between PGC-SCZ2 and GPC African and Latino ancestry cohorts. For varying P-value thresholds (*P*_T_), the number of SNPs, fraction of these with the same direction of effect, and significance of a one-sided binomial test (*P*_sign_).

| *P*_T_ | | PGC-trained | | | GPC-trained | | |
| --- | --- | --- | --- | --- | --- | --- | --- |
|  |  | African (admixed) | African (>75%) | Latino | African (admixed) | African (>75%) | Latino |
| 5×10^-8^ | SNPs | 112 | 111 | 114 | 0 | 1 | 1 |
|  | fraction | 0.679 | 0.61 | 0.640 | NaN | 1 | 1 |
|  | *P*_sign_ | **9.91×10^-5^** | 0.0111 | 0.00175 | 0.5 | 0.5 | 0.5 |
| 5×10^-7^ | SNPs | 209 | 205 | 215 | 1 | 2 | 1 |
|  | fraction | 0.646 | 0.58 | 0.665 | 1 | 0.5 | 1 |
|  | *P*_sign_ | **1.47×10^-5^** | 0.0126 | **7.33×10^-7^** | 0.5 | 0.750 | 0.5 |
| 5×10^-6^ | SNPs | 456 | 446 | 474 | 19 | 12 | 15 |
|  | fraction | 0.616 | 0.567 | 0.665 | 0.526 | 0.667 | 0.533 |
|  | *P*_sign_ | **3.94×10^-7^** | 0.00258 | **3.38×10^-13^** | 0.5 | 0.194 | 0.5 |
| 5×10^-5^ | SNPs | 1127 | 1092 | 1176 | 148 | 136 | 142 |
|  | fraction | 0.571 | 0.551 | 0.651 | 0.507 | 0.515 | 0.535 |
|  | *P*_sign_ | **1.22×10^-6^** | 0.000387 | **8.80×10^-26^** | 0.467 | 0.399 | 0.225 |
| 5×10^-4^ | SNPs | 3049 | 2943 | 3310 | 1255 | 1175 | 996 |
|  | fraction | 0.553 | 0.533 | 0.602 | 0.531 | 0.529 | 0.533 |
|  | *P*_sign_ | **2.14×10^-9^** | 0.000174 | **1.76×10^-32^** | 0.0159 | 0.0271 | 0.0197 |
| 0.005 | SNPs | 10451 | 9963 | 12003 | 8870 | 8616 | 6893 |
|  | fraction | 0.536 | 0.524 | 0.557 | 0.539 | 0.526 | 0.548 |
|  | *P*_sign_ | **9.26×10^-14^** | **1.24×10^-6^** | **1.87×10^-36^** | **9.07×10^-14^** | **9.05×10^-7^** | **1.08×10^-15^** |
| 0.05 | SNPs | 40189 | 37762 | 48806 | 55336 | 54628 | 42522 |
|  | fraction | 0.518 | 0.512 | 0.531 | 0.523 | 0.517 | 0.531 |
|  | *P*_sign_ | **2.03×10^-13^** | **9.66×10^-7^** | **3.78×10^-44^** | **5.00×10^-27^** | **5.66×10^-15^** | **4.57×10^-37^** |

###

### **Supplemental Table 5.** Binomial sign test for consistency of directions of allelic effect between GPC African and Latino ancestry cohorts. For varying P-value thresholds (*P*_T_), the number of SNPs, fraction of these with the same direction of effect, and significance of a one-sided binomial test (*P*_sign_).

| *P*_T_ | | African (admixed) | African (>75%) | Latino | |
| --- | --- | --- | --- | --- | --- |
|  |  |  |  | African (admixed) | African (>75%) |
| 5×10^-8^ | SNPs | 0 | 1 | 1 | 1 |
|  | fraction | NaN | 0 | 0 | 0 |
|  | *P*_sign_ | 0.5 | 1 | 1 | 1 |
| 5×10^-7^ | SNPs | 2 | 1 | 1 | 1 |
|  | fraction | 1 | 0 | 0 | 0 |
|  | *P*_sign_ | 0.250 | 1 | 1 | 1 |
| 5×10^-6^ | SNPs | 19 | 14 | 15 | 15 |
|  | fraction | 0.526 | 0.429 | 0.600 | 0.667 |
|  | *P*_sign_ | 0.500 | 0.788 | 0.304 | 0.151 |
| 5×10^-5^ | SNPs | 189 | 173 | 155 | 149 |
|  | fraction | 0.54 | 0.497 | 0.568 | 0.57 |
|  | *P*_sign_ | 0.154 | 0.560 | 0.0539 | 0.0505 |
| 5×10^-4^ | SNPs | 1621 | 1526 | 1187 | 1144 |
|  | fraction | 0.506 | 0.492 | 0.515 | 0.524 |
|  | *P*_sign_ | 0.327 | 0.739 | 0.162 | 0.0519 |
| 0.005 | SNPs | 11389 | 11115 | 8111 | 7810 |
|  | fraction | 0.514 | 0.513 | 0.516 | 0.516 |
|  | *P*_sign_ | 0.00184 | 0.00235 | 0.00257 | 0.00225 |
| 0.05 | SNPs | 71756 | 70890 | 49948 | 47601 |
|  | fraction | 0.51 | 0.507 | 0.502 | 0.504 |
|  | *P*_sign_ | **4.59×10^-8^** | **7.32×10^-5^** | 0.157 | 0.0450 |

###

### **Supplemental Table 6. Association of cross-ancestry and meta-analysis polygenic scores with schizophrenia.** For each training dataset, *P_T_* is the *P*-value threshold applied to its results; *R*^2^ and *R*^2^_liability_ are the variance explained by a given score in terms of Nagelkerke’s *R*^2^ and on the liability scale, respectively; *β* and *SE* are the regression coefficient and its standard error.

| Training set | *P*_T_ | African  (6152 cases, 3918 controls) | | | |  | Latino  (1234 cases, 3090 controls) | | | |  | European  (6046 cases, 4534 controls)* | | | |
| --- | --- | --- | --- | --- | --- | --- | --- | --- | --- | --- | --- | --- | --- | --- | --- |
|  |  | *R*^2^ | *R*^2^ _liability_ | *β* (SE) | *P* |  | *R*^2^ | *R*^2^ _liability_ | *β* (SE) | *P* |  | *R*^2^ | *R*^2^ _liability_ | *β* (SE) | *P* |
| African | 5×10^-5^ | 0.000 | 0.000 | -0.004  (0.023) | 0.845 |  | 0.001 | 0.000 | 0.081  (0.044) | 0.0657 |  | 0.000 | 0.000 | 0.044  (0.030) | 0.143 |
|  | 5×10^-4^ | 0.003 | 0.001 | 0.036  (0.008) | **6.04×10^-6^** |  | 0.001 | 0.000 | 0.027  (0.018) | 0.130 |  | 0.000 | 0.000 | 0.012  (0.011) | 0.311 |
|  | 0.005 | 0.015 | 0.008 | 0.028  (0.003) | **1.04×10^-25^** |  | 0.001 | 0.000 | 0.016  (0.007) | 0.0294 |  | 0.001 | 0.000 | 0.011  (0.005) | 0.0230 |
|  | 0.05 | 0.021 | 0.011 | 0.011  (0.001) | **2.42×10^-35^** |  | 0.003 | 0.001 | 0.011  (0.003) | 0.00123 |  | 0.001 | 0.001 | 0.009  (0.003) | 0.000395 |
|  | 0.5 | 0.025 | 0.013 | 0.007  (0.0005) | **3.47×10^-41^** |  | 0.002 | 0.001 | 0.006  (0.002) | 0.00545 |  | 0.002 | 0.001 | 0.007  (0.002) | 0.000154 |
|  |  |  |  |  |  |  |  |  |  |  |  |  |  |  |  |
| Latino | 5×10^-5^ | 0.000 | 0.000 | 0.013  (0.016) | 0.421 |  | 0.000 | 0.000 | 0.024  (0.027) | 0.365 |  | 0.000 | 0.001 | 0.038  (0.020) | 0.0613 |
|  | 5×10^-4^ | 0.000 | 0.000 | 0.005  (0.006) | 0.365 |  | 0.004 | 0.003 | 0.035  (0.010) | 0.000299 |  | 0.000 | 0.000 | 0.012  (0.008) | 0.164 |
|  | 0.005 | 0.001 | 0.001 | 0.004  (0.002) | 0.0374 |  | 0.010 | 0.008 | 0.019  (0.003) | **7.41×10^-9^** |  | 0.002 | 0.005 | 0.016  (0.003) | **5.76×10^-6^** |
|  | 0.05 | 0.001 | 0.002 | 0.002  (0.001) | 0.00513 |  | 0.021 | 0.016 | 0.010  (0.001) | **1.33×10^-15^** |  | 0.004 | 0.007 | 0.008  (0.002) | **1.62×10^-8^** |
|  | 0.5 | 0.001 | 0.001 | 0.001  (0.0005) | 0.0278 |  | 0.027 | 0.020 | 0.006  (0.001) | **3.11×10^-19^** |  | 0.004 | 0.008 | 0.006  (0.001) | **1.60×10^-9^** |
|  |  |  |  |  |  |  |  |  |  |  |  |  |  |  |  |
| PGC-SCZ2 | 5×10^-5^ | 0.007 | 0.004 | 0.141  (0.019) | **1.04×10^-13^** |  | 0.047 | 0.011 | 0.624  (0.052) | **1.49×10^-33^** |  | 0.060 | 0.025 | 0.665  (0.035) | **8.97×10^-80^** |
|  | 5×10^-4^ | 0.010 | 0.005 | 0.109  (0.013) | **8.02×10^-18^** |  | 0.048 | 0.011 | 0.411  (0.034) | **2.34×10^-34^** |  | 0.072 | 0.030 | 0.481  (0.023) | **2.02×10^-95^** |
|  | 0.005 | 0.011 | 0.006 | 0.071  (0.008) | **6.12×10^-20^** |  | 0.059 | 0.013 | 0.276  (0.020) | **2.07×10^-41^** |  | 0.079 | 0.033 | 0.293  (0.014) | **5.67×10^-104^** |
|  | 0.05 | 0.011 | 0.005 | 0.041  (0.005) | **8.25×10^-19^** |  | 0.075 | 0.017 | 0.181  (0.012) | **9.02×10^-52^** |  | 0.084 | 0.035 | 0.172  (0.008) | **4.03×10^-110^** |
|  | 0.5 | 0.011 | 0.006 | 0.031  (0.003) | **5.98×10^-20^** |  | 0.062 | 0.014 | 0.119  (0.009) | **1.34×10^-43^** |  | 0.072 | 0.030 | 0.107  (0.005) | **8.64×10^-95^** |
|  |  |  |  |  |  |  |  |  |  |  |  |  |  |  |  |
| Meta-analysis | 5×10^-5^ | 0.009 | 0.005 | 0.163  (0.020) | **8.92×10^-16^** |  | 0.047 | 0.011 | 0.673  (0.055) | **7.24×10^-34^** |  | 0.063 | 0.026 | 0.743  (0.038) | **2.15×10^-84^** |
|  | 5×10^-4^ | 0.018 | 0.009 | 0.158  (0.014) | **3.24×10^-30^** |  | 0.064 | 0.015 | 0.527  (0.038) | **1.04×10^-44^** |  | 0.076 | 0.032 | 0.546  (0.026) | **1.77×10^-100^** |
|  | 0.005 | 0.026 | 0.014 | 0.118  (0.008) | **7.77×10^-44^** |  | 0.081 | 0.019 | 0.361  (0.023) | **3.95×10^-55^** |  | 0.089 | 0.037 | 0.363  (0.016) | **1.32×10^-115^** |
|  | 0.05 | 0.032 | 0.017 | 0.076  (0.005) | **5.46×10^-53^** |  | 0.089 | 0.021 | 0.227  (0.014) | **1.10×10^-59^** |  | 0.088 | 0.037 | 0.220  (0.010) | **1.73×10^-114^** |
|  | 0.5 | 0.032 | 0.017 | 0.053  (0.003) | **4.37×10^-53^** |  | 0.081 | 0.019 | 0.158  (0.010) | **1.56×10^-55^** |  | 0.079 | 0.033 | 0.151  (0.007) | **3.50×10^-104^** |

*Analyses of PGC-SCZ2 and meta-analysis scores utilized an independent cohort of European ancestry GPC participants (4094 cases, 3888 controls).

### **Supplemental Table 7. Association results for polygenic scores based on varying levels of linkage disequilibrium among SNPs.** For each training dataset, based on *P_T_* > 0.5, LD is the *r*^2^ value threshold applied during the clumping procedure; *R*^2^ and *R*^2^ _liability_ are the variance explained by a given score in terms of Nagelkerke’s *R*^2^ and on the liability scale, respectively; *β* and *SE* are the regression coefficient and its standard error; and *P* is its significance.

| Training set | LD (*r^2^*) | African  (6152 cases, 3918 controls) | | | |  | Latino  1234 cases, 3090 controls) | | | |  | European  (6046 cases, 4534 controls) | | | |
| --- | --- | --- | --- | --- | --- | --- | --- | --- | --- | --- | --- | --- | --- | --- | --- |
|  |  | *R*^2^ | *R*^2^ _liability_ | *β* (SE) | *P* |  | *R*^2^ | *R*^2^ _liability_ | *β* (SE) | *P* |  | *R*^2^ | *R*^2^ _liability_ | *β* (SE) | *P* |
| African | 0.1 | 0.025 | 0.013 | 0.007  (0.0005) | **3.47×10^-41^** |  | 0.002 | 0.001 | 0.006  (0.002) | 0.00545 |  | 0.002 | 0.001 | 0.007  (0.002) | **0.000154** |
|  | 0.5 | 0.024 | 0.013 | 0.002  (0.0002) | **3.60×10^-40^** |  | 0.004 | 0.001 | 0.003  (0.001) | **0.00063** |  | 0.003 | 0.001 | 0.004  (0.001) | **1.96×10^-6^** |
|  | 0.8 | 0.023 | 0.012 | 0.001  (0.0001) | **5.70×10^-39^** |  | 0.004 | 0.001 | 0.002  (0.001) | **0.000184** |  | 0.003 | 0.002 | 0.002  (0.0004) | **2.35×10^-7^** |
|  |  |  |  |  |  |  |  |  |  |  |  |  |  |  |  |
| Latino | 0.1 | 0.001 | 0.001 | 0.001  (0.0005) | 0.0278 |  | 0.027 | 0.020 | 0.006  (0.001) | **3.11×10^-19^** |  | 0.004 | 0.008 | 0.006  (0.001) | **1.60×10^-9^** |
|  | 0.5 | 0.001 | 0.001 | 0.0005  (0.0002) | 0.0104 |  | 0.029 | 0.021 | 0.002  (0.0002) | **1.45×10^-20^** |  | 0.006 | 0.011 | 0.003 (0.0004) | **1.37×10^-12^** |
|  | 0.8 | 0.001 | 0.002 | 0.0003 (0.0001) | 0.008 |  | 0.029 | 0.021 | 0.001  (0.0001) | **6.12×10^-21^** |  | 0.006 | 0.013 | 0.002  (0.0002) | **3.35×10^-14^** |
|  |  |  |  |  |  |  |  |  |  |  |  |  |  |  |  |
| PGC-SCZ2 | 0.1 | 0.011 | 0.006 | 0.031  (0.003) | **5.98×10^-20^** |  | 0.062 | 0.014 | 0.119  (0.009) | **1.34×10^-43^** |  | 0.072 | 0.030 | 0.107  (0.005) | **8.64×10^-95^** |
|  | 0.5 | 0.016 | 0.008 | 0.013 (0.001) | **7.03×10^-28^** |  | 0.082 | 0.019 | 0.049 (0.003) | **1.17×10^-55^** |  | 0.092 | 0.039 | 0.046  (0.002) | **1.55×10^-119^** |
|  | 0.8 | 0.019 | 0.010 | 0.008 (0.001) | **3.10×10^-32^** |  | 0.089 | 0.021 | 0.026 (0.002) | **7.51×10^-60^** |  | 0.097 | 0.041 | 0.026 (0.001) | **1.65×10^-125^** |
|  |  |  |  |  |  |  |  |  |  |  |  |  |  |  |  |
| Meta-analysis | 0.1 | 0.032 | 0.017 | 0.053  (0.003) | **4.37×10^-53^** |  | 0.081 | 0.019 | 0.158  (0.010) | **1.56×10^-55^** |  | 0.079 | 0.033 | 0.151  (0.007) | **3.50×10^-104^** |
|  | 0.5 | 0.035 | 0.018 | 0.020 (0.001) | **7.72×10^-58^** |  | 0.105 | 0.025 | 0.063  (0.004) | **1.09×10^-69^** |  | 0.097 | 0.041 | 0.061 (0.003) | **5.77×10^-126^** |
|  | 0.8 | 0.036 | 0.018 | 0.012 (0.001) | **1.04×10^-58^** |  | 0.106 | 0.025 | 0.032  (0.002) | **8.88×10^-70^** |  | 0.099 | 0.042 | 0.032 (0.001) | **1.61×10^-127^** |

###

**Supplemental Table 8. Improvements in polygenic prediction by incorporating scores based on multiple ancestries.** For each training dataset, *P_T_* is the *P*-value threshold, *LogLik* is the log likelihood for the combined model including a given score, *Δ_LogLik_* is the difference in L*ogLik* compared to a model including only the PGC-trained score and covariates, **𝜒^2^** is the chi-square for the model improvement, and *P*_logLik_ is its significance; *R*^2^ and *R*^2^_liability_ give the incremental variance explained in terms of Nagelkerke’s *R*^2^ and on the liability scale.

| Training set(s) | *P*_T_ | African  (6152 cases, 3918 controls) | | | | | |  | Latino  (1234 cases, 3090 controls) | | | | | |  | European  (4094 cases, 3888 controls) | | | | | |
| --- | --- | --- | --- | --- | --- | --- | --- | --- | --- | --- | --- | --- | --- | --- | --- | --- | --- | --- | --- | --- | --- |
|  |  | *LogLik* | *Δ_LogLik_* | **𝜒^2^** | *P_LogLik_* | *R*^2^ | *R*^2^ _liability_ |  | *LogLik* | *Δ_LogLik_* | **𝜒^2^** | *P_LogLik_* | *R*^2^ | *R*^2^ _liability_ |  | *LogLik* | *Δ_LogLik_* | **𝜒^2^** | *P* | *R*^2^ | *R*^2^ _liability_ |
| African | 5×10^-5^ | -6629.28 | 0.01 | 0.02 | 0.891 | 0.000 | 0.000 |  | -2344.96 | 1.15 | 2.30 | 0.129 | 0.001 | 0.000 |  | -5123.37 | 0.11 | 0.23 | 0.633 | 0.000 | 0.000 |
|  | 5×10^-4^ | -6609.62 | 10.14 | 20.28 | **6.71×10^-6^** | 0.003 | 0.001 |  | -2343.57 | 0.45 | 0.91 | 0.341 | 0.000 | 0.000 |  | -5081.46 | 0.07 | 0.14 | 0.710 | 0.000 | 0.000 |
|  | 0.005 | -6560.49 | 54.31 | 108.63 | **1.96×10^-25^** | 0.014 | 0.006 |  | -2324.60 | 1.21 | 2.43 | 0.119 | 0.001 | 0.000 |  | -5055.92 | 1.76 | 3.52 | 0.0607 | 0.001 | 0.000 |
|  | 0.05 | -6538.68 | 78.80 | 157.61 | **3.78×10^-36^** | 0.021 | 0.009 |  | -2294.54 | 2.95 | 5.89 | 0.015 | 0.002 | 0.001 |  | -5036.15 | 4.45 | 8.90 | 0.00286 | 0.001 | 0.001 |
|  | 0.5 | -6520.84 | 93.98 | 187.97 | **8.84×10^-43^** | 0.025 | 0.011 |  | -2317.38 | 2.28 | 4.55 | 0.033 | 0.001 | 0.001 |  | -5074.85 | 8.26 | 16.52 | **4.82×10^-5^** | 0.002 | 0.001 |
|  |  |  |  |  |  |  |  |  |  |  |  |  |  |  |  |  |  |  |  |  |  |
| Latino | 5×10^-5^ | -6628.95 | 0.34 | 0.69 | 0.407 | 0.000 | 0.000 |  | -2345.97 | 0.13 | 0.27 | 0.607 | 0.000 | 0.000 |  | -5122.56 | 0.93 | 1.85 | 0.174 | 0.000 | 0.000 |
|  | 5×10^-4^ | -6619.43 | 0.33 | 0.67 | 0.415 | 0.000 | 0.000 |  | -2339.05 | 4.97 | 9.94 | 0.00162 | 0.003 | 0.001 |  | -5081.37 | 0.16 | 0.32 | 0.572 | 0.000 | 0.000 |
|  | 0.005 | -6613.15 | 1.66 | 3.32 | 0.0683 | 0.000 | 0.000 |  | -2312.87 | 12.94 | 25.88 | **3.63×10^-7^** | 0.008 | 0.004 |  | -5052.05 | 5.64 | 11.27 | 0.000787 | 0.002 | 0.001 |
|  | 0.05 | -6614.73 | 2.75 | 5.51 | 0.0189 | 0.001 | 0.000 |  | -2269.97 | 27.51 | 55.03 | **1.19×10^-13^** | 0.016 | 0.009 |  | -5034.14 | 6.46 | 12.92 | 0.000326 | 0.002 | 0.001 |
|  | 0.5 | -6613.18 | 1.64 | 3.29 | 0.0698 | 0.000 | 0.000 |  | -2282.74 | 36.92 | 73.83 | **8.50×10^-18^** | 0.021 | 0.011 |  | -5073.02 | 10.09 | 20.17 | **7.08×10^-6^** | 0.003 | 0.001 |
|  |  |  |  |  |  |  |  |  |  |  |  |  |  |  |  |  |  |  |  |  |  |
| African and Latino | 5×10^-5^ | -6628.94 | 0.35 | 0.71 | 0.702 | 0.000 | 0.000 |  | -2344.83 | 1.28 | 2.56 | 0.278 | 0.001 | 0.000 |  | -5122.46 | 1.03 | 2.07 | 0.356 | 0.000 | 0.000 |
|  | 5×10^-4^ | -6609.26 | 10.50 | 21.00 | **2.75×10^-5^** | 0.003 | 0.001 |  | -2338.62 | 5.40 | 10.80 | 0.00451 | 0.003 | 0.002 |  | -5081.30 | 0.23 | 0.46 | 0.795 | 0.000 | 0.000 |
|  | 0.005 | -6558.99 | 55.82 | 111.63 | **5.74×10^-25^** | 0.015 | 0.006 |  | -2311.76 | 14.06 | 28.12 | **7.85×10^-7^** | 0.008 | 0.005 |  | -5050.43 | 7.25 | 14.50 | 0.000710 | 0.002 | 0.001 |
|  | 0.05 | -6536.36 | 81.12 | 162.23 | **5.92×10^-36^** | 0.021 | 0.009 |  | -2267.52 | 29.97 | 59.93 | **9.69×10^-14^** | 0.017 | 0.010 |  | -5029.89 | 10.71 | 21.41 | **2.24×10^-5^** | 0.003 | 0.001 |
|  | 0.5 | -6519.65 | 95.17 | 190.33 | **4.67×10^-42^** | 0.025 | 0.011 |  | -2281.31 | 38.35 | 76.69 | **2.22×10^-17^** | 0.022 | 0.012 |  | -5065.23 | 17.88 | 35.76 | **1.72×10^-8^** | 0.005 | 0.002 |

### **Supplemental Table 9. Association of cross-ancestry and meta-analysis polygenic scores with bipolar disorder.** For each training dataset, *P_T_* is the *P*-value threshold applied to its results; *R*^2^ and *R*^2^_liability_ are the variance explained by a given score in terms of Nagelkerke’s *R*^2^ and on the liability scale, respectively; *β* and *SE* are the regression coefficient and its standard error.

| Training set | *P*_T_ | African  (1766 cases, 2535 controls) | | | |  | Latino  (1032 cases, 3090 controls) | | | |  | European  (2626 cases, 4093 controls)* | | | |
| --- | --- | --- | --- | --- | --- | --- | --- | --- | --- | --- | --- | --- | --- | --- | --- |
|  |  | *R*^2^ | *R*^2^ _liability_ | *β* (SE) | *P* |  | *R*^2^ | *R*^2^ _liability_ | *β* (SE) | *P* |  | *R*^2^ | *R*^2^ _liability_ | *β* (SE) | *P* |
| African | 5×10^-5^ | 0.000 | 0.000 | 0.005 (0.034) | 0.884 |  | 0.000 | 0.000 | 0.026 (0.048) | 0.582 |  | 0.000 | 0.000 | 0.045 (0.037) | 0.221 |
|  | 5×10^-4^ | 0.006 | 0.002 | 0.053 (0.012) | 5.17**×10^-6^** |  | 0.001 | 0.000 | 0.035 (0.019) | 0.0669 |  | 0.000 | 0.000 | 0.007 (0.014) | 0.619 |
|  | 0.005 | 0.033 | 0.008 | 0.038 (0.004) | 1.90**×10^-23^** |  | 0.002 | 0.001 | 0.019 (0.008) | 0.0152 |  | 0.000 | 0.000 | 0.005 (0.006) | 0.374 |
|  | 0.05 | 0.040 | 0.010 | 0.014 (0.001) | 1.05**×10^-26^** |  | 0.004 | 0.001 | 0.012 (0.004) | 0.000945 |  | 0.000 | 0.000 | 0.004 (0.003) | 0.151 |
|  | 0.5 | 0.044 | 0.010 | 0.008 (0.001) | 3.81**×10^-28^** |  | 0.005 | 0.001 | 0.009 (0.002) | 0.000152 |  | 0.000 | 0.000 | 0.003 (0.002) | 0.166 |
|  |  |  |  |  |  |  |  |  |  |  |  |  |  |  |  |
| Latino | 5×10^-5^ | 0.000 | 0.000 | 0.006 (0.024) | 0.821 |  | 0.001 | 0.000 | 0.046 (0.029) | 0.113 |  | 0.001 | 0.000 | 0.043 (0.025) | 0.0802 |
|  | 5×10^-4^ | 0.000 | 0.000 | 0.008 (0.008) | 0.331 |  | 0.003 | 0.001 | 0.031 (0.010) | 0.00292 |  | 0.000 | 0.000 | -0.007 (0.010) | 0.477 |
|  | 0.005 | 0.002 | 0.001 | 0.008 (0.003) | 0.0143 |  | 0.006 | 0.002 | 0.016 (0.004) | 1.86**×10^-5^** |  | 0.001 | 0.001 | 0.011 (0.004) | 0.00639 |
|  | 0.05 | 0.001 | 0.000 | 0.002 (0.001) | 0.0667 |  | 0.016 | 0.004 | 0.009 (0.001) | 1.61**×10^-11^** |  | 0.002 | 0.001 | 0.006 (0.002) | 0.00117 |
|  | 0.5 | 0.000 | 0.000 | 0.000 (0.001) | 0.717 |  | 0.019 | 0.005 | 0.006 (0.001) | 2.20**×10^-13^** |  | 0.002 | 0.001 | 0.004 (0.001) | 0.000795 |
|  |  |  |  |  |  |  |  |  |  |  |  |  |  |  |  |
| PGC-SCZ2 | 5×10^-5^ | 0.005 | 0.001 | 0.111 (0.029) | 9.81**×10^-5^** |  | 0.023 | 0.007 | 0.446 (0.054) | 1.93**×10^-16^** |  | 0.020 | 0.008 | 0.408 (0.039) | 1.93**×10^-25^** |
|  | 5×10^-4^ | 0.006 | 0.002 | 0.087 (0.019) | 5.24**×10^-6^** |  | 0.021 | 0.006 | 0.278 (0.035) | 3.41**×10^-15^** |  | 0.019 | 0.008 | 0.259 (0.025) | 2.74**×10^-24^** |
|  | 0.005 | 0.004 | 0.001 | 0.044 (0.012) | 0.000221 |  | 0.025 | 0.007 | 0.184 (0.021) | 7.98**×10^-18^** |  | 0.027 | 0.011 | 0.179 (0.015) | 1.45**×10^-33^** |
|  | 0.05 | 0.004 | 0.001 | 0.025 (0.007) | 0.000410 |  | 0.029 | 0.009 | 0.114 (0.012) | 1.54**×10^-20^** |  | 0.029 | 0.012 | 0.104 (0.008) | 5.56**×10^-36^** |
|  | 0.5 | 0.006 | 0.002 | 0.022 (0.005) | 1.47**×10^-5^** |  | 0.021 | 0.006 | 0.071 (0.009) | 1.01**×10^-15^** |  | 0.026 | 0.011 | 0.067 (0.006) | 3.63**×10^-32^** |
|  |  |  |  |  |  |  |  |  |  |  |  |  |  |  |  |
| Meta-analysis | 5×10^-5^ | 0.007 | 0.002 | 0.146 (0.030) | 1.65**×10^-6^** |  | 0.028 | 0.008 | 0.530 (0.058) | 4.26**×10^-20^** |  | 0.022 | 0.009 | 0.467 (0.043) | 1.18**×10^-27^** |
|  | 5×10^-4^ | 0.012 | 0.003 | 0.130 (0.021) | 3.11**×10^-10^** |  | 0.031 | 0.009 | 0.372 (0.039) | 1.05**×10^-21^** |  | 0.023 | 0.010 | 0.313 (0.028) | 4.84**×10^-29^** |
|  | 0.005 | 0.023 | 0.006 | 0.109 (0.013) | 4.40**×10^-18^** |  | 0.038 | 0.011 | 0.252 (0.024) | 3.19**×10^-26^** |  | 0.032 | 0.013 | 0.226 (0.017) | 3.32**×10^-39^** |
|  | 0.05 | 0.029 | 0.008 | 0.069 (0.007) | 9.97**×10^-22^** |  | 0.044 | 0.013 | 0.162 (0.014) | 8.75**×10^-30^** |  | 0.035 | 0.015 | 0.142 (0.010) | 4.26**×10^-42^** |
|  | 0.5 | 0.031 | 0.008 | 0.049 (0.005) | 2.98**×10^-23^** |  | 0.035 | 0.010 | 0.104 (0.010) | 1.67**×10^-24^** |  | 0.031 | 0.013 | 0.098 (0.008) | 1.45**×10^-37^** |

## *Analyses of PGC-SCZ2 and meta-analysis scores utilized an independent cohort of European ancestry GPC participants (2172 cases, 2209 controls).

### **Supplemental Table 10. Improved fine-mapping resolution at 9 established schizophrenia loci by trans-ancestry meta-analysis of PGC-SCZ2 and GPC-Latino.** For each index SNP, descriptives of 99% credible sets constructed from PGC-SCZ2 and meta-analysis results are displayed; credible sets are summarized in terms of genomic coordinates, number of SNPs, and length of the genomic interval in kilobases (kb), and improvement in fine-mapping resolution is given in terms of reductions in the number of SNPs and corresponding interval length.

| Index SNP | Chr | 99% credible set: PGC-SCZ2 | | |  | 99% credible set: Meta-analysis | | |  | 99% credible set: reduction | |
| --- | --- | --- | --- | --- | --- | --- | --- | --- | --- | --- | --- |
|  |  | Location (GRCh37) | SNPs | Interval (kb) |  | Location (GRCh37) | SNPs | Interval (kb) |  | SNPs | Interval (kb) |
| rs6670165 | 1 | 177,247,854- 177,300,809 | 22 | 52.96 |  | 177,247,854- 177,299,241 | 16 | 51.39 |  | 6 | 1.57 |
| rs7432375 | 3 | 135,872,958- 136,500,733 | 68 | 627.78 |  | 135,992,645- 136,500,733 | 66 | 508.09 |  | 2 | 119.69 |
| rs4391122 | 5 | 60,513,566- 60,843,706 | 42 | 330.14 |  | 60,545,515- 60,843,706 | 32 | 298.19 |  | 10 | 31.95 |
| chr7_2025096_I | 7 | 1,982,181- 2,050,401 | 37 | 68.22 |  | 1,982,181- 2,048,220 | 23 | 66.04 |  | 14 | 2.18 |
| rs211829 | 7 | 110,039,196- 110,097,308 | 20 | 58.11 |  | 110,039,196- 110,091,388 | 19 | 52.19 |  | 1 | 5.92 |
| chr11_46350213_D | 11 | 46,343,189- 46,684,677 | 98 | 341.49 |  | 46,343,189- 46,673,344 | 81 | 330.16 |  | 17 | 11.33 |
| rs9922678 | 16 | 9,885,027- 9,960,879 | 95 | 75.852 |  | 9,886,831- 9,960,879 | 84 | 74.05 |  | 11 | 1.8 |
| rs12691307 | 16 | 29,924,422- 30,013,674 | 45 | 89.252 |  | 29,927,702- 30,013,674 | 42 | 85.97 |  | 3 | 3.28 |
| rs6002655 | 22 | 42,375,761- 42,689,370 | 60 | 313.61 |  | 42,382,748- 42,689,370 | 44 | 306.62 |  | 16 | 6.99 |

###

### **Supplemental Table 11.** **Improved fine-mapping resolution at 12 established schizophrenia loci by trans-ancestry meta-analysis of PGC-SCZ2, GPC-AA, and GPC-Latino.** For each index SNP, descriptives of 99% credible sets constructed from PGC-SCZ2 and meta-analysis results are displayed; credible sets are summarized in terms of genomic coordinates, number of SNPs, and length of the genomic interval in kilobases (kb), and improvement in fine-mapping resolution is given in terms of reductions in the number of SNPs and corresponding interval length.

| Index SNP | Chr | 99% credible set: PGC-SCZ2 | | |  | 99% credible set: Meta-analysis | | |  | 99% credible set: reduction | |
| --- | --- | --- | --- | --- | --- | --- | --- | --- | --- | --- | --- |
|  |  | Location (GRCh37) | SNPs | Interval (kb) |  | Location (GRCh37) | SNPs | Interval (kb) |  | SNPs | Interval (kb) |
| rs6670165 | 1 | 177,247,854- 177,300,809 | 22 | 52.96 |  | 177,247,854- 177,276,006 | 7 | 28.15 |  | 15 | 24.80 |
| chr2_146436222_I | 2 | 146,419,047- 146,441,828 | 22 | 22.78 |  | 146,419,047- 146,440,672 | 17 | 21.63 |  | 5 | 1.16 |
| rs2909457 | 2 | 162,798,581- 162,891,848 | 28 | 93.27 |  | 162,798,581- 162,845,855 | 20 | 47.27 |  | 8 | 45.99 |
| rs4391122 | 5 | 60,513,566- 60,843,706 | 42 | 330.14 |  | 60,545,515- 60,843,706 | 31 | 298.19 |  | 11 | 31.95 |
| rs111294930 | 5 | 152,098,705- 152,323,236 | 104 | 224.53 |  | 152,100,732- 152,323,236 | 98 | 222.5 |  | 6 | 2.03 |
| chr7_2025096_I | 7 | 1,982,181- 2,050,401 | 37 | 68.22 |  | 1,982,181- 2,048,220 | 23 | 66.04 |  | 14 | 2.18 |
| rs3735025 | 7 | 137,042,224- 137,085,250 | 52 | 43.03 |  | 137,047,137- 137,085,250 | 44 | 38.11 |  | 8 | 4.91 |
| chr11_46350213_D | 11 | 46,343,189- 46,684,677 | 98 | 341.49 |  | 46,368,241- 46,673,344 | 52 | 305.1 |  | 46 | 36.39 |
| rs12903146 | 15 | 61,831,680- 61,887,768 | 36 | 56.09 |  | 61,831,863- 61,873,251 | 29 | 41.39 |  | 7 | 14.70 |
| rs56873913 | 19 | 50,067,508- 50,103,252 | 34 | 35.74 |  | 50,078,276- 50,103,252 | 24 | 24.98 |  | 10 | 10.77 |
| chr22_39987017_D | 22 | 39,975,307- 40,015,493 | 17 | 40.19 |  | 39,975,691- 40,015,493 | 12 | 39.8 |  | 5 | 0.38 |
| rs9607782 | 22 | 41,418,154- 41,627,775 | 18 | 209.62 |  | 41,414,055- 41,617,897 | 17 | 203.84 |  | 1 | 5.78 |

##

###

## **Supplemental Figures**

### **Supplemental Figure 1.** PCA clustering of GPC subjects with super populations from the 1000 Genomes Project.


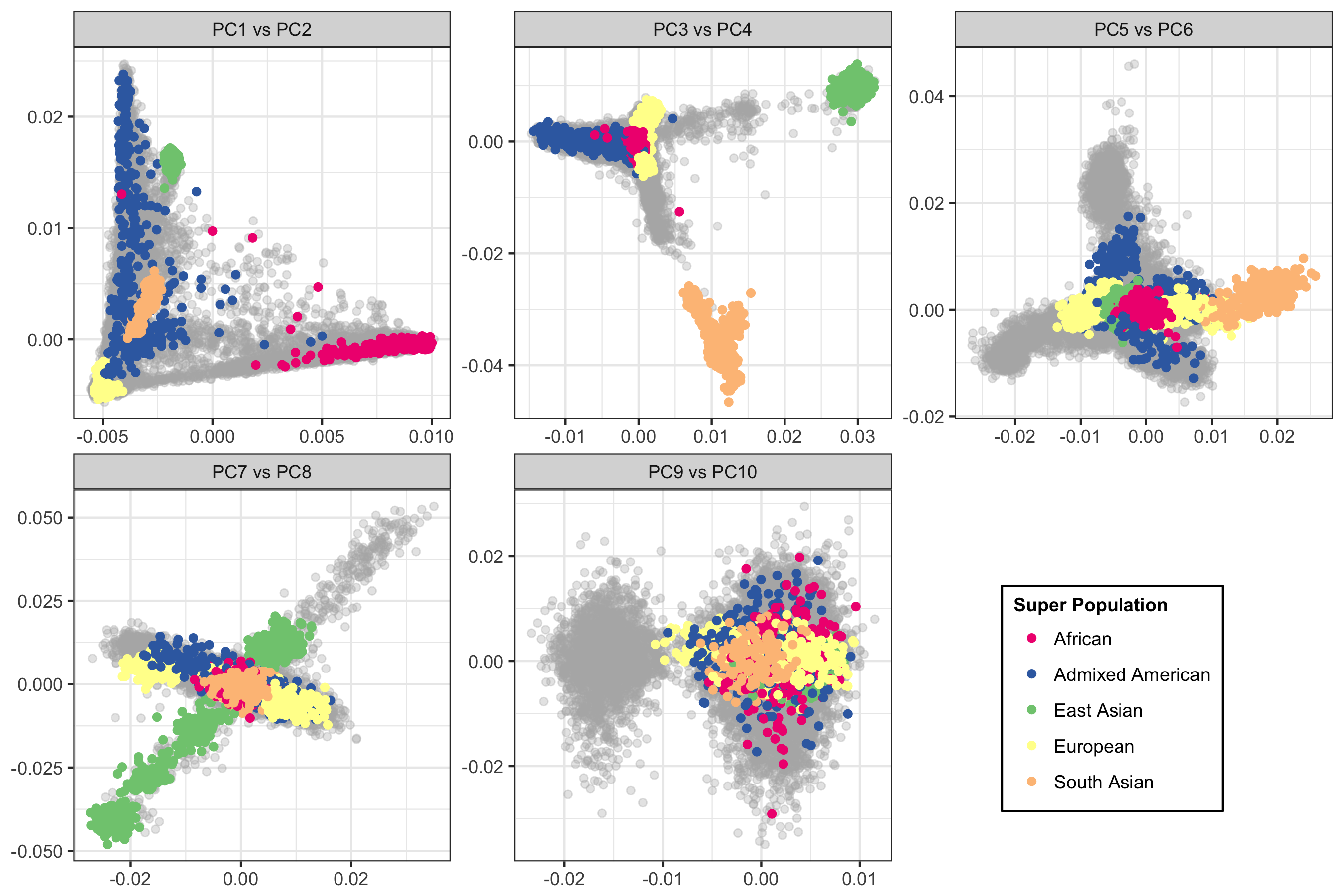


###

### **Supplemental Figure 2.** PCA clustering of GPC subjects with African ancestry populations from the 1000 Genomes Project.


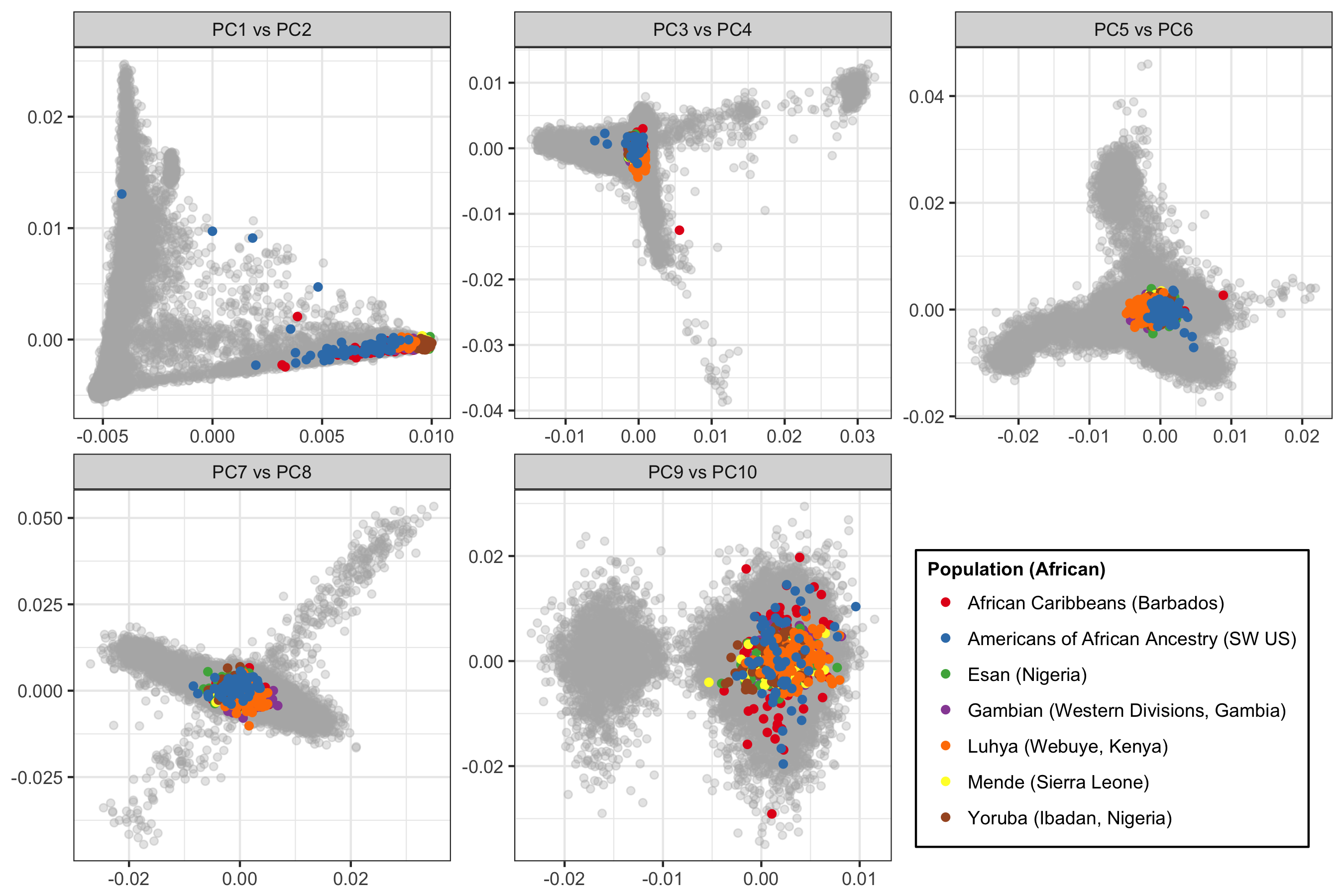


### **Supplemental Figure 3.** PCA clustering of GPC subjects with Admixed American ancestry populations from the 1000 Genomes Project.


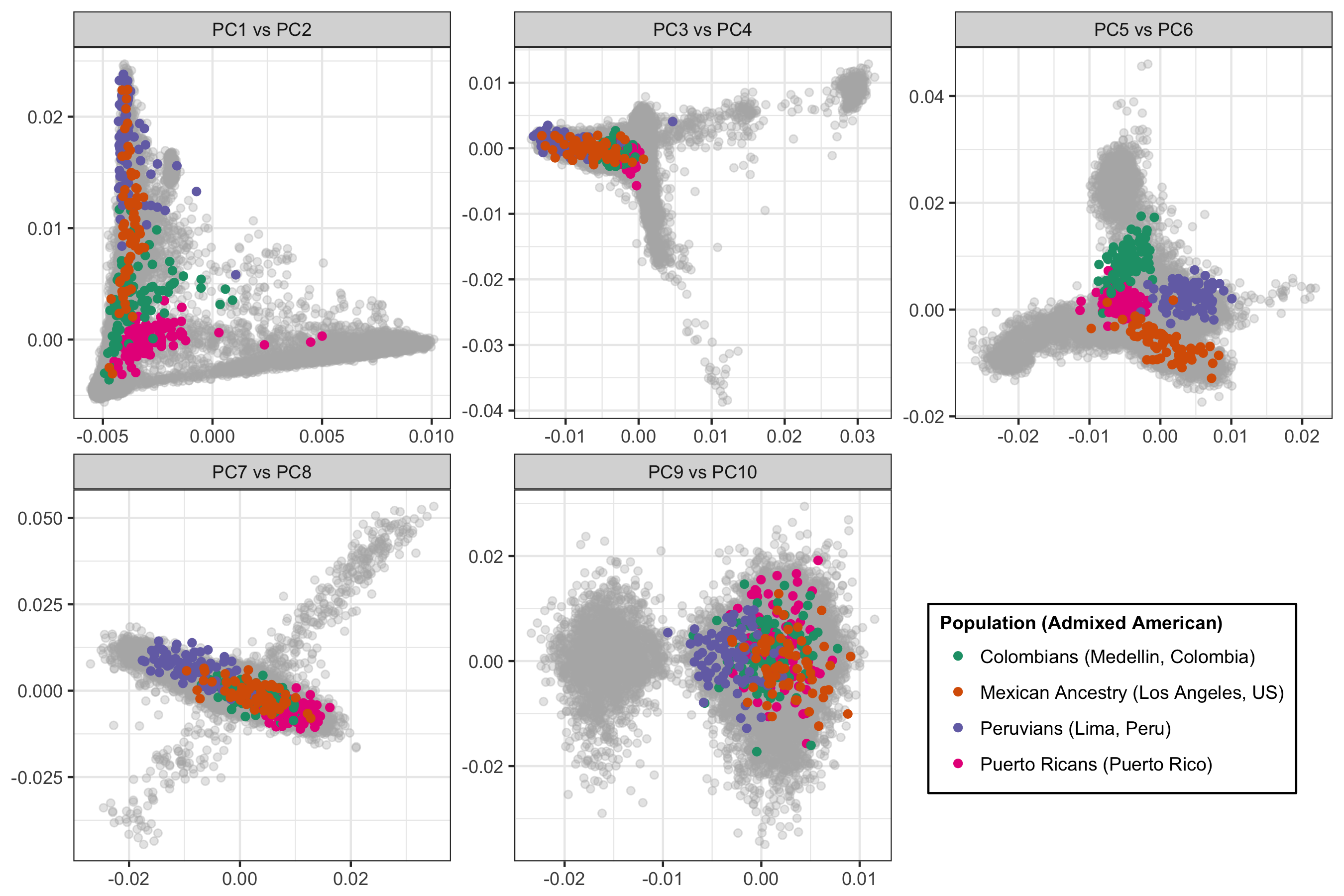


###

### **Supplemental Figure 4. Manhattan plots for primary within-ancestry GWAS of schizophrenia in GPC-AA and GPC-Latino.** Red and blue dashed lines denote thresholds for genome-wide significance (*P*<5×10^-8^) and replication follow-up in PGC-SCZ2 (*P*<10^-6^).


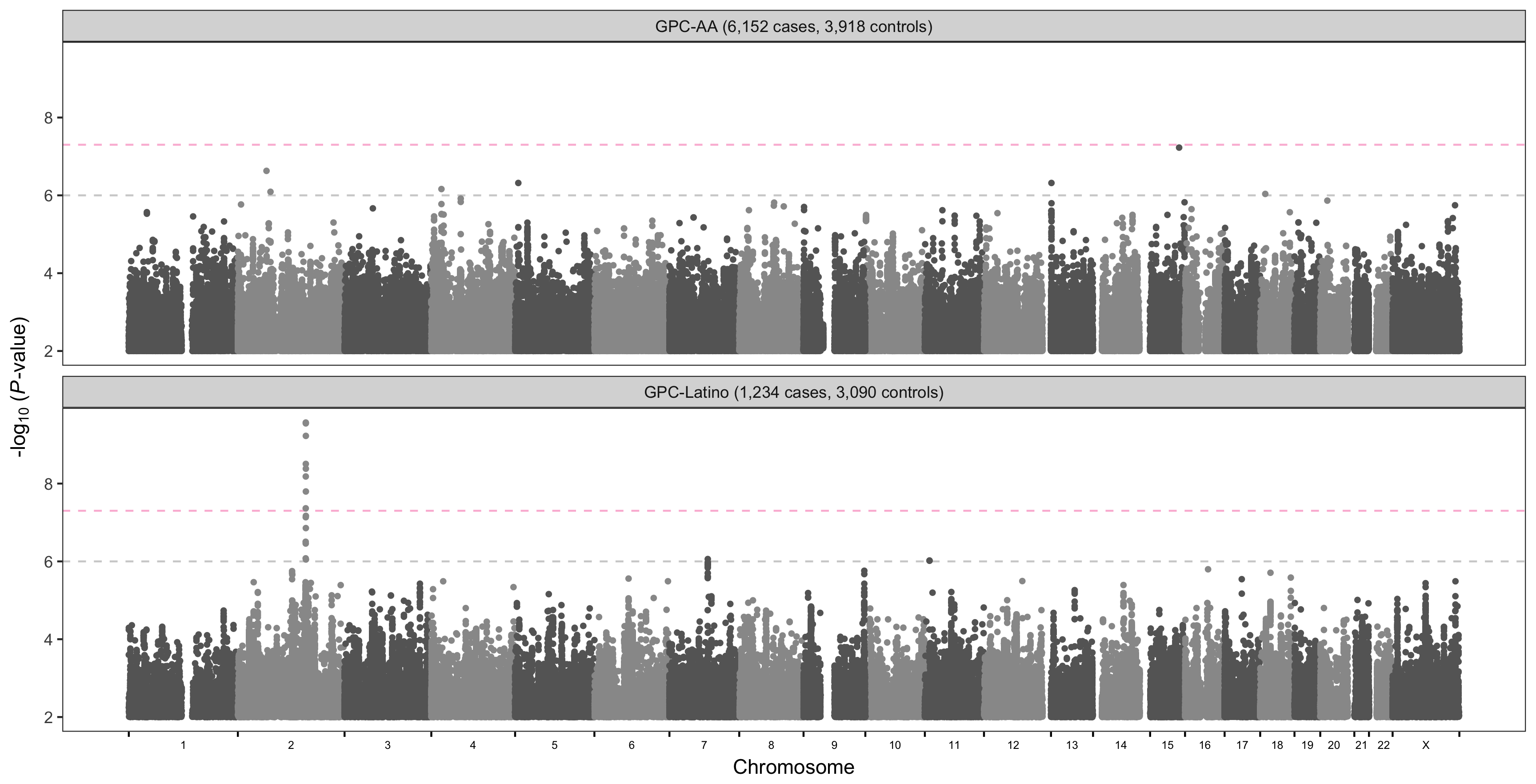


###

### **Supplemental Figure 5. Quantile-quantile (QQ) plots for primary within-ancestry GWAS of schizophrenia.** For analyses of GPC-AA (*left*) and GPC-Latino (*right*), observed distributions of test-statistics are plotted against expected distributions under the null, in terms of as the negative base-10 logarithm of the association *P-*value. The genomic control factor, lambda (λ), and lambda scaled to a sample size of 1,000 cases and 1,000 controls (λ_1000_) are displayed.

**
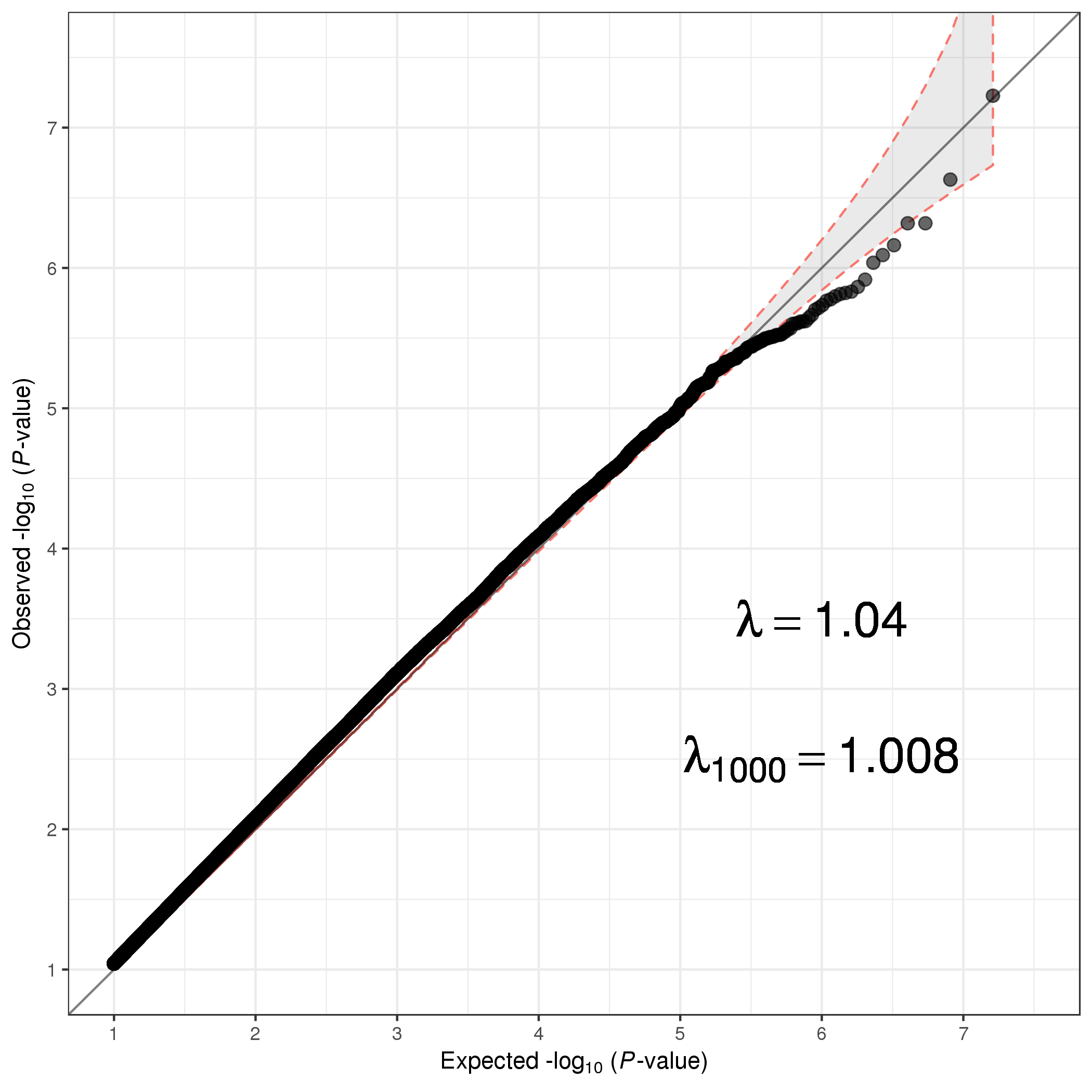

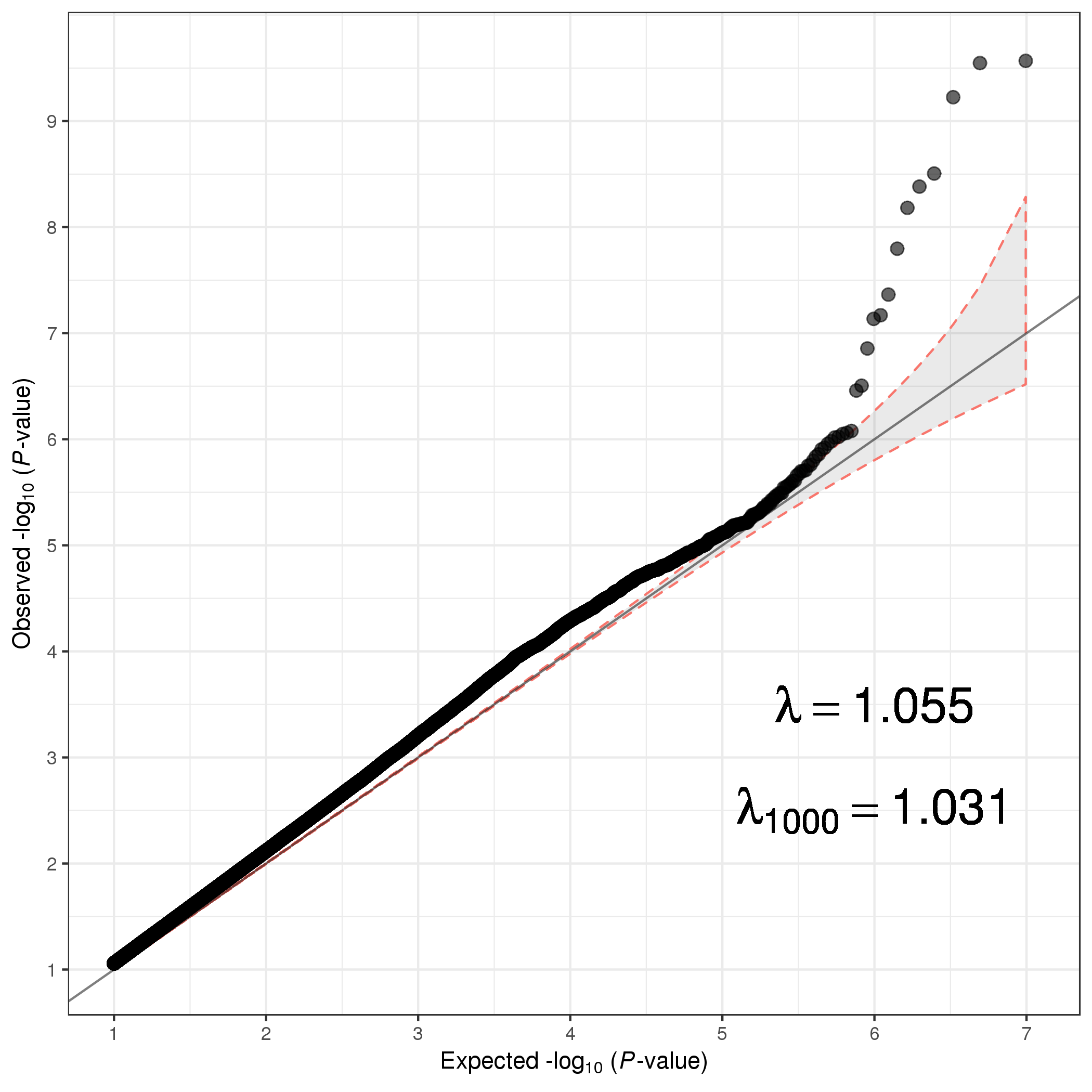
**

**Supplemental Figure 6.** Novel association of *GALNT13* SNPs observed in Latino cases and controls (*upper*) Regional association plot for the novel association detected in the GPC-Latino GWAS of schizophrenia. The strength of LD of each SNP with the “index” SNP, displayed as a large purple diamond, is indicated by its color. Plot was created using the LocusZoom [(11)](https://paperpile.com/c/pLC4Y0/IBxWm) (<http://locuszoom.org/>). (lower) Forest plot of effect sizes in each wave of Latino data, and in the GPC-AA and PGC-SCZ2 results. Abbreviations are as follows: *frq_a* and *frq_u* denote the frequency of the reference allele among cases and controls, respectively; *or* and *se* are its odds ratio and its standard error; *p* is its significance; *cas* and *con* give the numbers of cases and controls; and *info* is the statistical imputation information as calculated by PLINK.


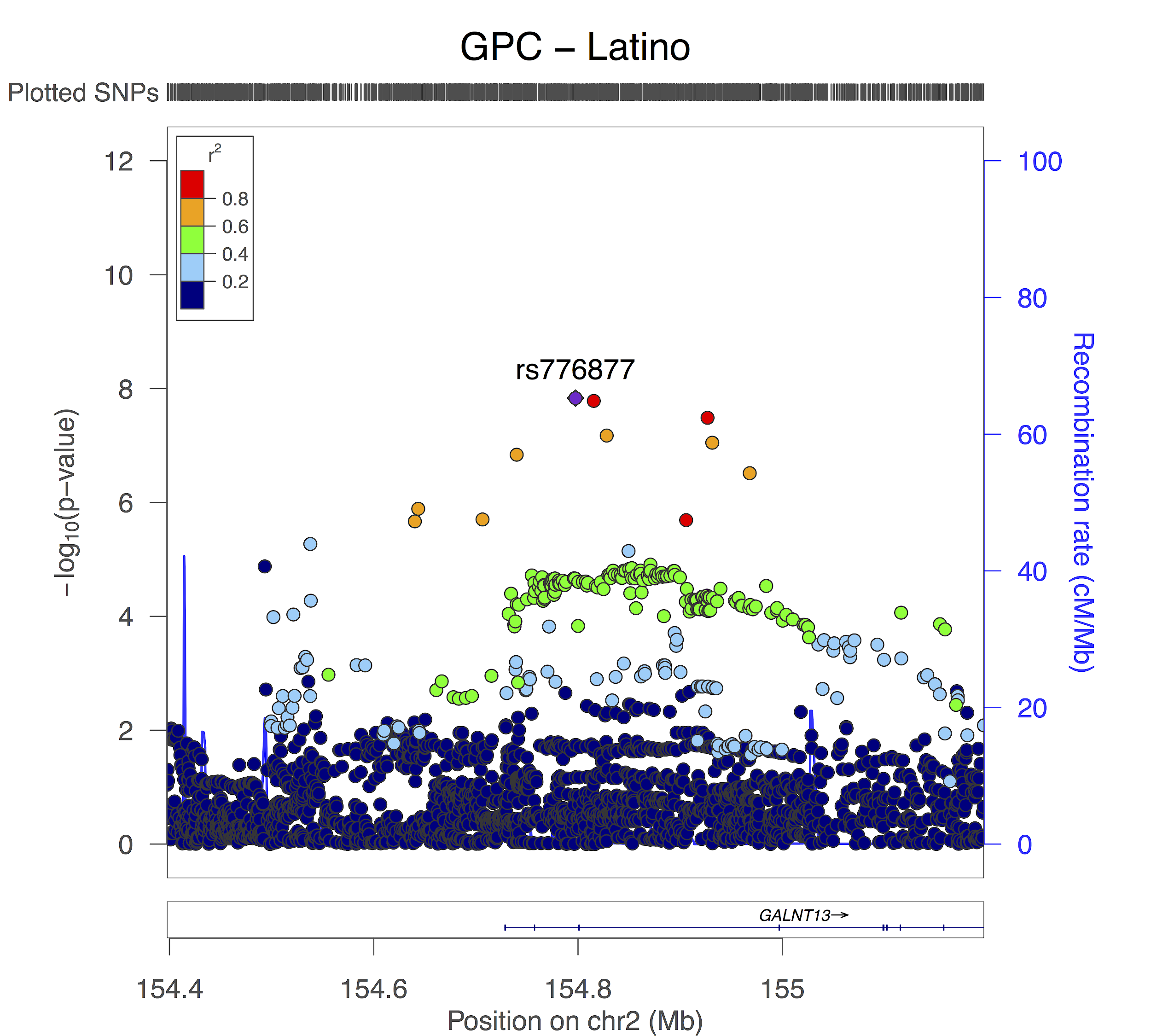

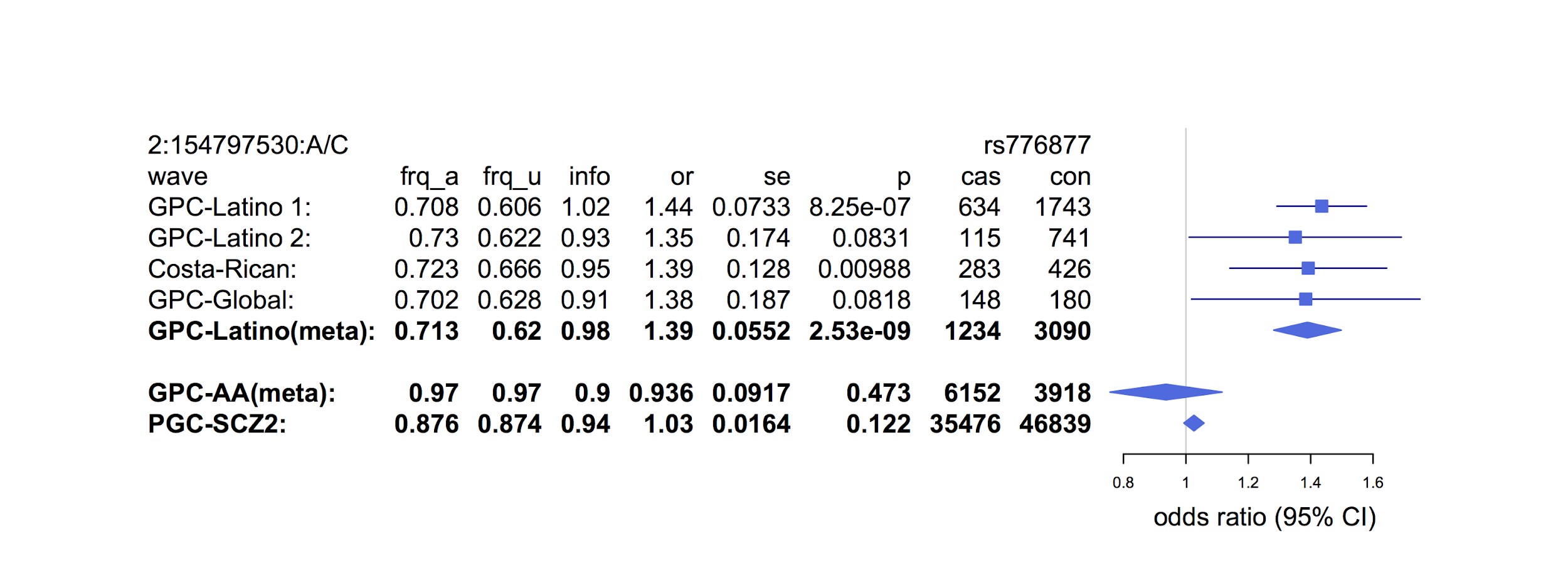


###

###

**Supplemental References**

1. [Castle DJ, Jablensky A, McGrath JJ, Carr V, Morgan V, Waterreus A, et al. The diagnostic interview for psychoses (DIP): development, reliability and applications. Psychol Med. 2006 Jan;36(1):69–80.](http://paperpile.com/b/pLC4Y0/ou9H)

2. [Nurnberger JI, Blehar MC, Kaufmann CA, York-Cooler C, Simpson SG, Harkavy-Friedman J, et al. Diagnostic Interview for Genetic Studies: Rationale, Unique Features, and Training. Arch Gen Psychiatry. 1994 Nov 1;51(11):849–59.](http://paperpile.com/b/pLC4Y0/8l39)

3. [American Psychiatric Association. Diagnostic and Statistical Manual of Mental Disorders, Fourth Edition, Text Revision (DSM-IV-TR). 4th ed. Vol. 1. Arlington, VA: American Psychiatric Association; 2000.](http://paperpile.com/b/pLC4Y0/aaAu)

4. [World Health Organization. The ICD-10 Classification of Mental and Behavioural Disorders: Diagnostic Criteria for Research. World Health Organization; 1993. 248 p.](http://paperpile.com/b/pLC4Y0/EfBt)

5. [American Psychiatric Association. Diagnostic and Statistical Manual of Mental Disorders (DSM-5®). American Psychiatric Pub; 2013. 991 p.](http://paperpile.com/b/pLC4Y0/L7oK)

6. [Sanders AR, Levinson DF, Duan J, Dennis JM, Li R, Kendler KS, et al. The Internet-based MGS2 control sample: self report of mental illness. Am J Psychiatry. 2010 Jul;167(7):854–65.](http://paperpile.com/b/pLC4Y0/kQBa)

7. [Shi J, Levinson DF, Duan J, Sanders AR, Zheng Y, Pe’er I, et al. Common variants on chromosome 6p22.1 are associated with schizophrenia. Nature. 2009 Aug 6;460(7256):753–7.](http://paperpile.com/b/pLC4Y0/4nKV)

8. [The GAIN Collaborative Research Group. New models of collaboration in genome-wide association studies: the Genetic Association Information Network. Nat Genet. 2007 Aug 29;39:1045.](http://paperpile.com/b/pLC4Y0/P0A0)

9. [Calkins ME, Dobie DJ, Cadenhead KS, Olincy A, Freedman R, Green MF, et al. The Consortium on the Genetics of Endophenotypes in Schizophrenia: model recruitment, assessment, and endophenotyping methods for a multisite collaboration. Schizophr Bull. 2007 Jan;33(1):33–48.](http://paperpile.com/b/pLC4Y0/FzED)

10. [Aliyu MH, Calkins ME, Swanson CL Jr, Lyons PD, Savage RM, May R, et al. Project among African-Americans to explore risks for schizophrenia (PAARTNERS): recruitment and assessment methods. Schizophr Res. 2006 Oct;87(1-3):32–44.](http://paperpile.com/b/pLC4Y0/Tve3)

11. [Pruim RJ, Welch RP, Sanna S, Teslovich TM, Chines PS, Gliedt TP, et al. LocusZoom: regional visualization of genome-wide association scan results. Bioinformatics. 2010;26:2336–7.](http://paperpile.com/b/pLC4Y0/IBxWm)
